# Identification of genes that are essential to restrict genome duplication to once per cell division

**DOI:** 10.18632/oncotarget.9008

**Published:** 2016-04-26

**Authors:** Alex Vassilev, Chrissie Y. Lee, Boris Vassilev, Wenge Zhu, Pinar Ormanoglu, Scott E. Martin, Melvin L. DePamphilis

**Affiliations:** ^1^ National Institute of Child Health and Human Development, National Institutes of Health, Bethesda, MD 20892-2753, USA; ^2^ Current address: NantBioscience, Culver City, CA 90232, USA; ^3^ Department of Biochemistry and Molecular Biology, George Washington University, Washington DC 20037, USA; ^4^ National Center of Advancing Translational Sciences, National Institutes of Health, Rockville, MD 20850, USA; ^5^ Current Address: Genentech, Inc., South San Francisco, CA 94080, USA

**Keywords:** DNA re-replication, endoreplication, cell cycle regulation, aneuploidy, polyploidy

## Abstract

Nuclear genome duplication is normally restricted to once per cell division, but aberrant events that allow excess DNA replication (EDR) promote genomic instability and aneuploidy, both of which are characteristics of cancer development. Here we provide the first comprehensive identification of genes that are essential to restrict genome duplication to once per cell division. An siRNA library of 21,584 human genes was screened for those that prevent EDR in cancer cells with undetectable chromosomal instability. Candidates were validated by testing multiple siRNAs and chemical inhibitors on both TP53+ and TP53- cells to reveal the relevance of this ubiquitous tumor suppressor to preventing EDR, and in the presence of an apoptosis inhibitor to reveal the full extent of EDR. The results revealed 42 genes that prevented either DNA re-replication or unscheduled endoreplication. All of them participate in one or more of eight cell cycle events. Seventeen of them have not been identified previously in this capacity. Remarkably, 14 of the 42 genes have been shown to prevent aneuploidy in mice. Moreover, suppressing a gene that prevents EDR increased the ability of the chemotherapeutic drug Paclitaxel to induce EDR, suggesting new opportunities for synthetic lethalities in the treatment of human cancers.

## INTRODUCTION

Genome instability is an integral part of cancer development. Cells isolated from human cancers typically vary widely in chromosomal content with both structural and numerical alterations, and they exhibit high rates of genome instability, as demonstrated by the rapid restoration of genomic heterogeneity following clonal selection [1]. In fact, genome instability is generally reported as the accumulation of polyploid and aneuploid cells [2]. Polyploid cells contain multiple copies of the entire genome. Aneuploid cells have either gained or lost entire chromosomes or parts of chromosomes. They exist in up to 80% of cancers, and they are associated with a poor prognosis for recovery [3]. Moreover, genome sequencing reveals that up to 37% of all tumors have transitioned through a polyploid state during their development [4], suggesting that tumorigenesis is accelerated by transition through the inherently unstable polyploid state. The increased potential of neoplastic cells to evolve more aggressive sub-clones also has been linked directly to genome instability [5, 6]. In fact, aneuploidy and resistance to chemotherapeutic drugs increase when polyploid cells are induced *in vitro* from cells derived from cancers or from non-transformed cells [3].

Human development requires trillions of cell divisions wherein nuclear DNA replication (S phase) is restricted to once per cell division by multiple regulatory pathways [7, 8]. Developmentally programmed endoreplication (a repeated S phase without an intervening mitosis or cytokinesis) is rare in mammals, although it occurs frequently in ferns, flowering plants, mollusks, arthropods, amphibians, and fish [9]. Two well characterized examples in mammals are the trophoblast giant cells required for embryo implantation and placentation, and the megakaryocytes required for platelet production [10]. Nevertheless, interruption of the mammalian cell division cycle by selective inhibition of specific genes can result in excess nuclear DNA replication due either to unscheduled endoreplication or to DNA re-replication.

Antimitotic drugs, such as taxanes and vinca alkaloids, are useful cancer therapeutics, because they inhibit microtubule dynamics, thereby arresting proliferation when cells enter mitosis [11]. However, cells do not remain in mitosis indefinitely, because the anaphase-promoting complex (APC) is activated soon thereafter [12, 13]. Activation of the APC allows cells to re-enter G1 phase as tetraploid cells with either a single enlarged nucleus or several micronuclei [14]. This aberrant event is termed mitotic slippage, and it generally results in DNA damage and apoptosis. However, tetraploid cells, particularly those lacking a G1 checkpoint such as p53 or Rb deficient cancer cells, can proceed into S phase, thereby producing a single cell with a giant nucleus containing 8N DNA [15-17]. This constitutes ‘unscheduled endoreplication’, an event that can also occur by suppressing expression of genes that are either essential for cytokinesis [18] or for entrance into mitosis [19-22].

DNA re-replication occurs when the block to origin licensing is interrupted during S phase, and cells begin to re-replicate their nuclear DNA prior to completing S phase. This results in partially replicated chromatids that accumulate in giant nuclei ranging from 4N through 8N or even greater [23, 24]. Since DNA replication forks are sensitive to DNA damage, particularly in the form of double-stranded breaks, DNA re-replication induces DNA damage. Normal cells respond to DNA damage by arresting cell proliferation until the damage is repaired [25], whereas a robust DNA damage response in cancer cells elicits apoptosis [26, 27].

Anecdotal evidence suggests that genome instability arises when cells depend on fewer genes to prevent aberrant cell cycle events such as DNA re-replication, endoreplication, mitotic slippage, and acytokinesis. Normal cells contain multiple pathways that can prevent DNA re-replication [28], whereas cancer cells often depend on a single pathway to prevent excess DNA replication. For example, some cancer cells rely solely on geminin to prevent DNA re-replication dependent apoptosis [29, 30]. This would account for the fact that geminin is over-expressed in many tumors, and the prognosis for recovery is inversely related to the level of geminin expression [31, 32]. Moreover, suppressing geminin expression can prevent tumor growth [33].

Given these reports, we reasoned that the transition from a normal cell to a cancer cell must involve changes in the mechanisms that restrict genome duplication to once per cell division. In other words, fluctuations in the activity of a protein that prevents EDR could result in aneuploid or polyploid cells. For example, all four subunits of the chromosome passenger complex restrict genome duplication to once per cell division *in vitro*, and prevent aneuploidy/polyploidy during mouse development [34-37]. Thus, identification of the genes that are essential to prevent EDR in cancer cells would reveal the mechanisms that promote genomic stability by restricting genome duplication to once per cell division.

Previous efforts to identify such genes in genome-scale profiles of cell cycle regulators in HeLa [38] and U2OS [39] cells were incomplete. For example, neither study identified geminin, and only 10% of the results from these studies overlapped. Therefore, we set out to identify the most comprehensive collection of genes whose activity is essential to prevent EDR by screening siRNAs in a cancer cell line with undetectable chromosomal instability. Candidates from this screen should include the genes previously documented to be essential in preventing EDR, as well as genes that have not yet been reported in this capacity. The candidates were then subjected to a battery of validation assays in order to confirm the identity of genes essential to prevent EDR. The results revealed 42 genes that are associated with eight specific cell cycle events that restrict genome duplication to once per cell division; selective inhibition of any one of these genes induced EDR in cancer cells with an unusually stable genome.

## RESULTS

Genes essential to prevent EDR in human cancer cells were first selected from a high throughput screen (HTS) for siRNAs that induced the accumulation of cells with a nuclear DNA content greater than mitotic cells and then validated by a four-step protocol. First, raw data were analyzed using multiple statistical criteria to select genes for which at least two of the three tested siRNAs produced signals significantly above the mean. Second, each well of the genes selected by statistical analysis was further analyzed by constructing DNA histograms of the distribution of cells throughout the mitotic cell cycle. Third, interacting partners of the genes whose suppression produced the strongest signals were then tested in order to build confidence in the importance of a specific cell cycle event in preventing EDR. Fourth, a new siRNA was used to validate the efficacy of all candidate genes using standard laboratory transfection and fluorescence activated cell-sorting (FACS) protocols to quantify the extent of EDR.

**Figure 1:**
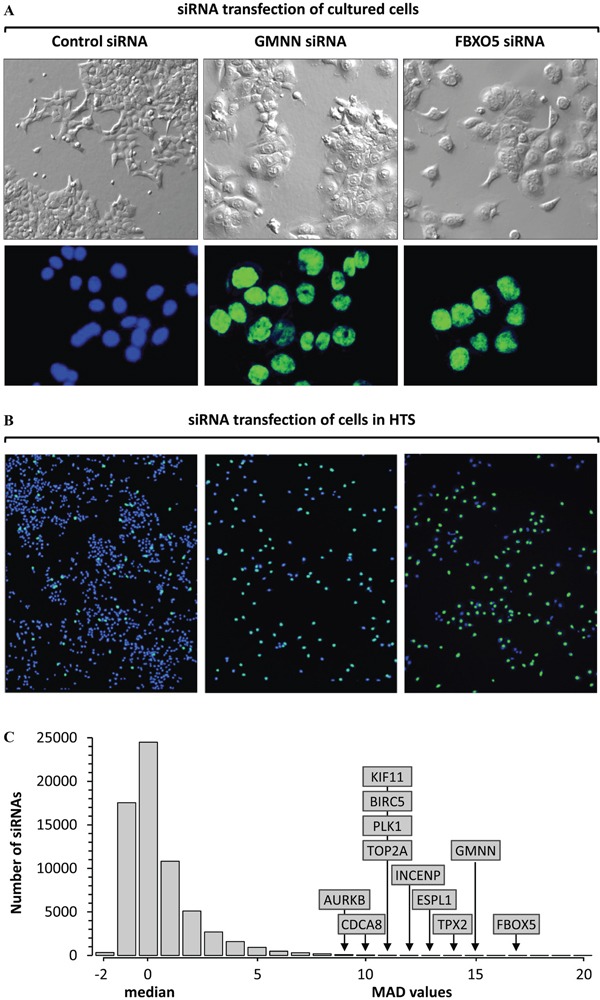
High throughput screen (HTS) for genes that prevent excess DNA replication **A.** Transfection of cultured HCT116 cells for 72 hours with Ambion's silent siRNA (control), or siGMNN/Geminin, or siFBXO5/EMI1 revealed a 10-fold increase in the fraction of cells with enlarged nuclei (58% with siGMNN; 49% with siFBXO5) relative to cells transfected with control (5%). Top panels are phase contrast images. Bottom panels are fluorescent images of cells stained with Hoechst 33342. Control nuclei are colored blue, and giant nuclei colored green. Images are all 20X magnification. **B.** HTS using the same siRNAs gave comparable results. Fluorescent images of nuclei in a single well treated with control siRNA revealed 95% of the nuclei contain ≤4N DNA (blue), and 5% of the nuclei contained >4N DNA (green). In contrast, 44% of the siGMNN treated cells and 51% of the siFBXO5 treated cells contained nuclei with >4N DNA (green). The total number of cells in control siRNA samples (blue plus green nuclei) was equivalent to cells in untreated wells (100%), whereas siGMNN treated wells contained 28% as many cells as control wells, and siFBXO5 treated wells contained 14% as many cells. **C.** The signals from 64,752 siRNAs in the Ambion primary screen were normalized to ‘silent siRNAs’ (0% signal) and siGMNN (100% signal) [43]. The fraction of cells with a nuclear DNA content >4N for the median of the three siRNAs in the primary HTS was compared with the median nuclear DNA content of the entire HTS. Subsequent validation assays confirmed that FBXO5, GMNN, TPX2, ESPL1, INCENP, PLK1, TOP2A, KIF11, BIRC5, CDCA8 and AURKB were critical for restriction of genome duplication to once per cell division. 5MAD was equivalent to ≥19.3% cells with >4N nuclear DNA, 10MAD was 33.4%, and 16MAD was 50.4%.

HCT116 cells were used in this screen, because they exhibit DNA damage–dependent and spindle-dependent checkpoints, and unlike most cancer cells, demonstrate no signs of chromosome instability [40]. Moreover, HCT116 cells are very sensitive to siRNA suppression of GMNN/Geminin [29] or FBXO5/Emi1 [41, 42], genes known to induce a robust accumulation of excess nuclear DNA in both TP53+ and TP53- cells. These results were confirmed for wild-type HCT116 cells under standard laboratory conditions (Figure 1A) and in the HTS (Figure 1B). In each case, the fraction of cells with enlarged nuclei was equivalent to the fraction of cells with >4N DNA content.

### Selecting candidate genes by statistical analysis

Each 384-well plate contained cells transfected with Ambion's ‘silent siRNAs’ that established the baseline, defined as 0% signal, and wells with cells transfected with siRNA against the Geminin gene (GMNN) that defined a 100% signal. The HTS data were then normalized to these controls in order to eliminate any plate-to-plate variation [43]. The fraction of cells with a nuclear DNA content >4N for the median of the three siRNAs in the primary HTS was compared with the median nuclear DNA content of the entire HTS (Figure 1C). The primary screen produced 454 genes with at least two siRNAs ≥3 median absolute deviation (MAD) above the median, of which 69 had at least two siRNAs ≥5MAD. Signals greater than 5MAD contain the most reproducibly active molecules [44]. Therefore, genes were selected from the ≥3MAD but <5MAD group that bore some relationship to one or more genes in the ≥5MAD group, as determined by GeneGo (MetaCore). These genes were combined with the ≥5MAD group and rescreened using four additional siRNAs per gene. The results from the primary and secondary screens yielded candidate genes with three to seven siRNAs ≥5MAD. To minimize the impact of off-target activities upon siRNA HTSs, ‘redundant siRNA analysis’ was applied by assigning a p-value to each gene based on how effectively all three of the siRNAs tested for that gene performed in the primary HTS [45]. Applying the criteria that candidates must have three or more siRNAs with an efficacy of ≥5MAD and that the log(p) for these siRNAs is greater than -2 yielded 59 candidates. Induction of DNA re-replication in cancer cells increases nuclear size and siRNAs against 11 of these genes increased the cell's nuclear area at least 70% as much as the 1.6-fold increase induced by siRNAs against either GMNN. Only 11 genes satisfied all these criteria: FBXO5 (7/7 siRNAs), GMNN (7/7), TPX2 (5/7), ESPL1 (7/7), INCENP (7/7), PLK1 (7/7), TOP2A (7/7), KIF11 (7/7), BIRC5 (4/7), CDCA8 (5/7) and AURKB (7/7) each had four to seven independent siRNAs that induced a significant fraction of cells (≥5MAD) to accumulate sufficient excess nuclear DNA (>4N) to form giant nuclei.

### Selecting candidate genes by nuclear DNA content analysis

**Figure 2:**
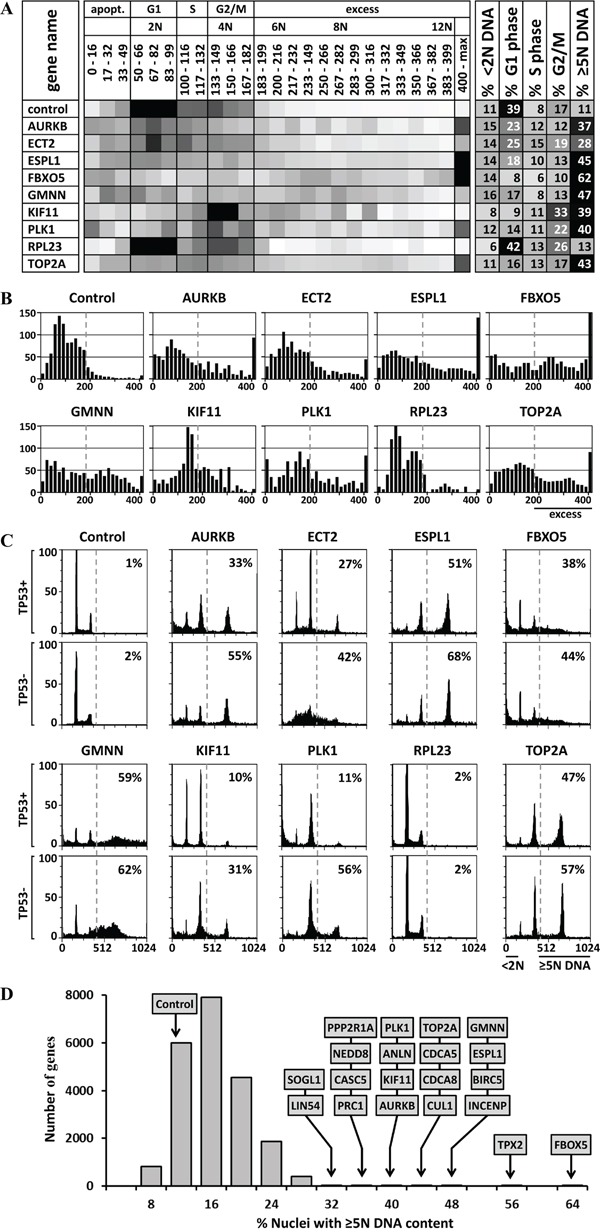
DNA content analysis and validation of HTS data **A.** Heat maps were created of the distribution of cells with a nuclear DNA content from <2N to >12N. Ten examples are shown; Supplementary Figure 1 contains the complete list of 85 candidate genes. Darker shading indicates higher fraction of cells with the indicated DNA content. The percentage of cells in G1 phase (2N DNA), S phase (>2N<4N DNA), and G2/M phase (4N DNA) was calculated, as well as the percentage of cells undergoing apoptosis (<2N DNA) or excess DNA replication (≥5N DNA). **B.** The data in panel A were expressed as histograms. **C.** Independent siRNAs against 8 candidate genes (AURKB, ECT2, ESPL1, FBXO5, GMNN, KIF11, PLK1 AND TOP2A), as well as one non-candidate gene (RPL23), and a control siRNA were validated in TP53+ and TP53- isogenic HCT116 cells using standard siRNA transfection and FACS protocols. Supplementary Figure 2 contains all the FACS profiles for the 42 validated genes. **D.** The 21,584 genes tested in the HTS were plotted according to the percentage of cells with ≥5N nuclear DNA content when depleted by the median effect siRNA in the HTS. The 20 genes with the greatest effect on preventing EDR are indicated. Of the cells treated with silent siRNA (control), 11% contained ≥5N nuclear DNA. These data revealed the fraction of cells with nuclear DNA content equivalent to G1 phase (2N), S phase (>2N<4N), G2/M phase (4N), apoptosis (<2N), or EDR (≥5N).

To determine the extent to which individual cells accumulated excess nuclear DNA, histograms of DNA content were constructed for each of the 64,752 siRNAs in the primary HTS. Since the final number of cells per well varied significantly, especially for genes whose depletion caused apoptosis, each histogram was extrapolated to represent 1000 cells per well. The fluorescence intensities were distributed into 25 intervals that covered the entire range of detected fluorescence. The results for each siRNA were then plotted as heat-maps in which the intensity of the color was proportional to the number of cells in the interval. Examples of the siRNAs with median effect for eight candidate genes, one negative gene, and the silent siRNA control are shown as heat maps (Figure 2A) and bar graphs (Figure 2B). In some cases, the position of nuclei with 2N DNA content (G1 phase cells) differed from the controls in the same plate. Differences in the amount of fluorescence per DNA unit that causes shifts in the DNA peaks positions of cell populations are routinely corrected during FACS by adjusting the laser PMT voltage of the instrument until the positions of the 2N or the 4N peak are the same for each sample [46]. Since the microplate cytometer used in the HTS did not make such corrections automatically, the data were corrected manually in order to ensure that cells with the same amount of DNA in each well have the same position in each histogram and the cell cycle phase distributions were calculated properly. If a shift in the positions of either the 2N (G1 phase) or 4N (G2/M phase) peaks could not be identified clearly in the histogram, then the siRNA was listed as a candidate for validation by standard FACS analysis. Supplementary Figure S1 contains heat maps of the median siRNA for all 96 candidate genes. These data allowed calculation of fraction of cells with nuclear DNA content equivalent to G1 phase (2N), S phase (>2N<4N), G2/M phase (4N), apoptosis (<2N), or EDR (≥5N). Quantifying the fraction of cells with a nuclear DNA content ≥5N ensured that only true EDR events were scored and not the trailing edge of cells accumulating in G2 or M phase.

### Validating candidate genes

Candidate genes were subjected to validation by transfecting cells under standard laboratory conditions with a new siRNA that did not overlap with any of the HTS siRNAs and then quantifying the DNA/cell by FACS analysis. The fraction of cells undergoing EDR was defined as those with a nuclear DNA content ≥5N and the fraction undergoing apoptosis as those with a nuclear DNA content <2N. The results were consistent with heat maps produced by the HTS (Figure 2C). Preliminary FACS data revealed that EDR signals <26% had only a 1/16 chance of being validated. This was due in part to the lower resolution scans used to generate the HTS DNA histograms, as reflected in lower MAD values. On that basis, 96 candidate genes were selected (Supplementary Figure S1). This group included those with highest statistical analysis scores (Figure 2D).

### TP53 can affect the extent of EDR

Since the tumor suppressor gene TP53 is directly involved in multiple pathways that can block cell cycle progression, promote apoptotic death and prevent tetraploid cells from undergoing EDR [47], we considered the possibility that the level of observed EDR might be affected by TP53 activity. To test this hypothesis, the ability of several candidate genes to prevent EDR was assayed in both HCT116(TP53+) and HCT116(TP53-) isogenic cells that differ only in expression of TP53. The results revealed that depletion of either PLK1 or KIF11 resulted in a three to five-fold increase in detectable EDR in the absence of TP53 (Figure 2C). The significant effect of TP53 expression prompted validation of all candidate genes in both TP53 positive and negative cells (Figures 4, 5, 6). The results revealed 12 genes for which EDR increased in HCT116(TP53-) cells by at least an additional 20% relative to HCT116(TP53+) cells (Supplementary Figure S3). Genes most affected by TP53 absence were PLK1 (+45%), CCNB1 (+40%), PRC1 (+32%), and RACGAP1 (+34%).

### Apoptosis can prevent detection of EDR

**Figure 3:**
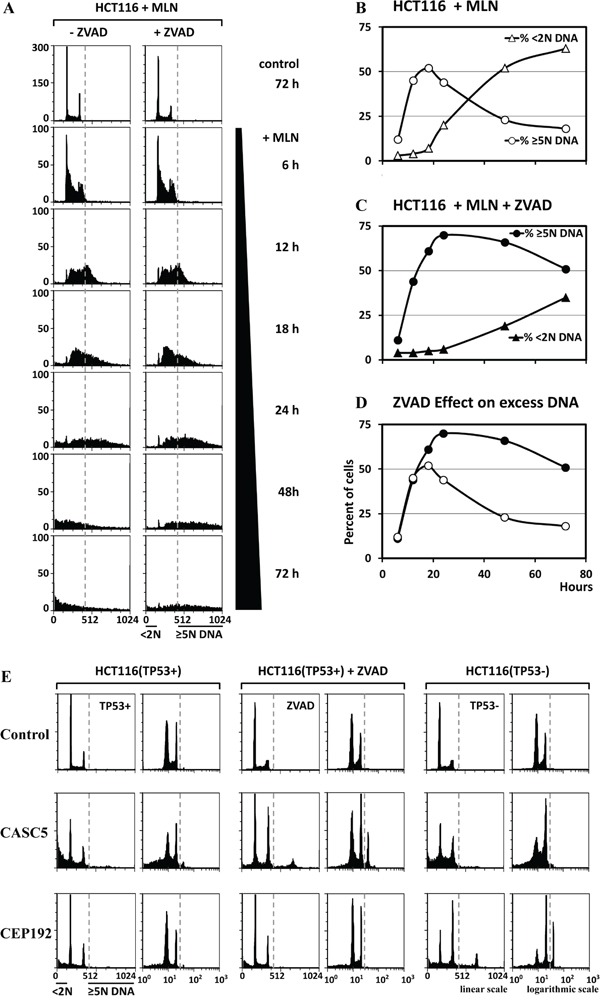
The EDR signal was increased when apoptosis was inhibited **A.** HCT116 cells were treated with 0.2μM MLN4924 (MLN), a specific inhibitor of neddylation, in either the presence or absence of 15μM ZVAD, a specific inhibitor of caspases. Cells were collected at the indicated times and subjected to FACS. The fraction of cells with ≥5N DNA content (representing EDR), and the fraction with <2N DNA content (representing apoptosis) were determined from the FACS data. **B.** The relationship between EDR and apoptosis as a function of time after addition of MLN is displayed in the absence (B) and in the presence (C) of ZVAD. **D.** In the presence of ZVAD, the rapid conversion of cells undergoing EDR into cells undergoing apoptosis 18 hours after addition of MLN was inhibited by ZVAD to reveal that 51% (not 18%) of the cells contain excess DNA. **E.** The results of the three validation assays (TP53+ cells, TP53+ cells +ZVAD, and TP53- cells) revealed that depletion of some genes (e.g. CASC5) induced EDR only when apoptosis was inhibited with ZVAD, while depletion of others (e.g. CEP192) induced EDR only in the absence of TP53.

**Figure 4:**
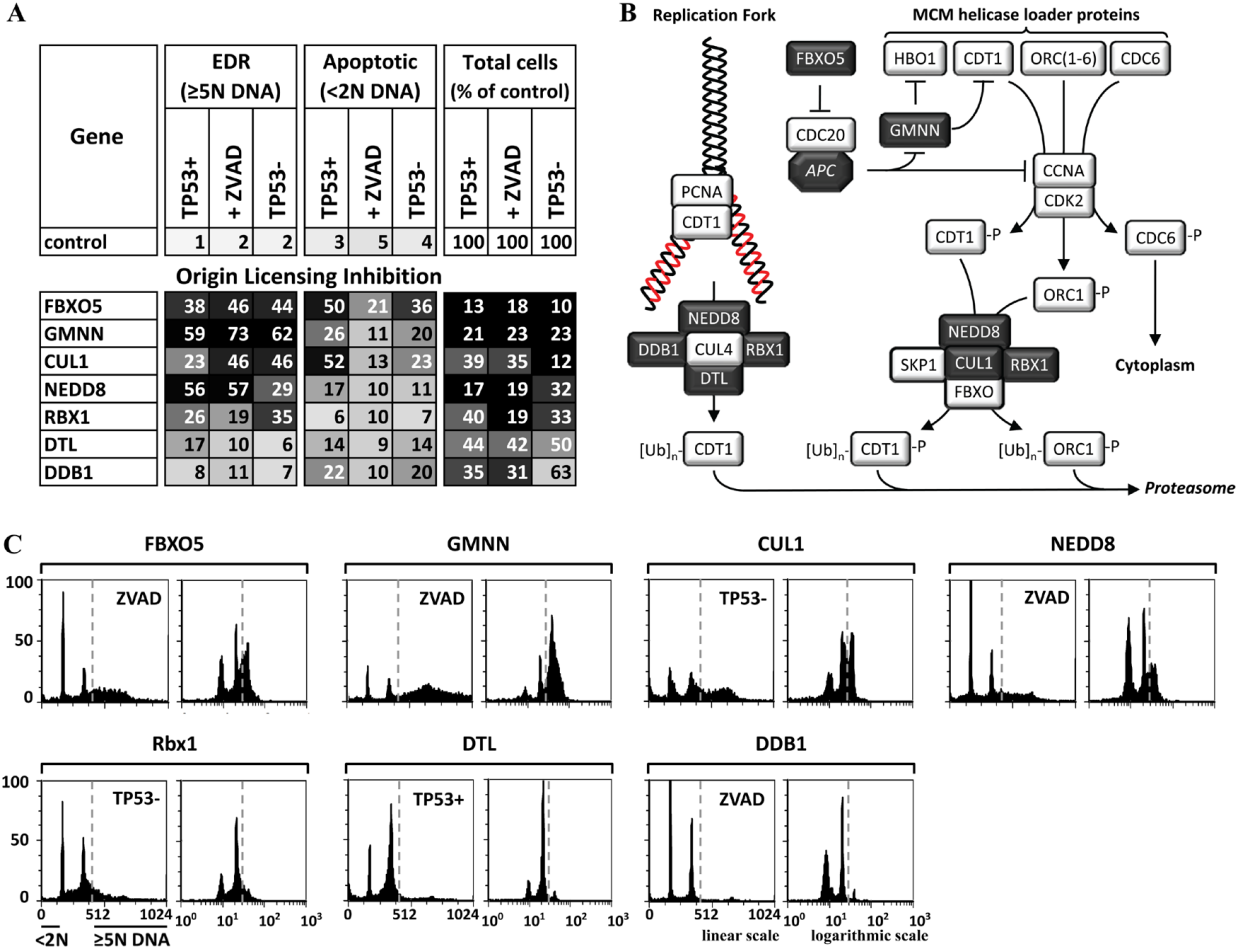
Genes that prevented premature origin licensing prevented DNA re-replication **A.** Seven of the validated genes have functions that prevent origin licensing during S and G2 phases of the cell cycle (illustrated in **B.** dark boxes with white font). The results of the three validation assays (TP53+ cells, TP53+ cells +ZVAD, and TP53- cells) carried out on each gene are summarized in panel A, and the FACS profiles are in Supplementary Figure S2A. FACS profiles for the validation assay with the strongest EDR signal for five of these genes are in panel **C.** The FACS profiles are consistent with induction of DNA re-replication when one of these genes is depleted.

**Figure 5:**
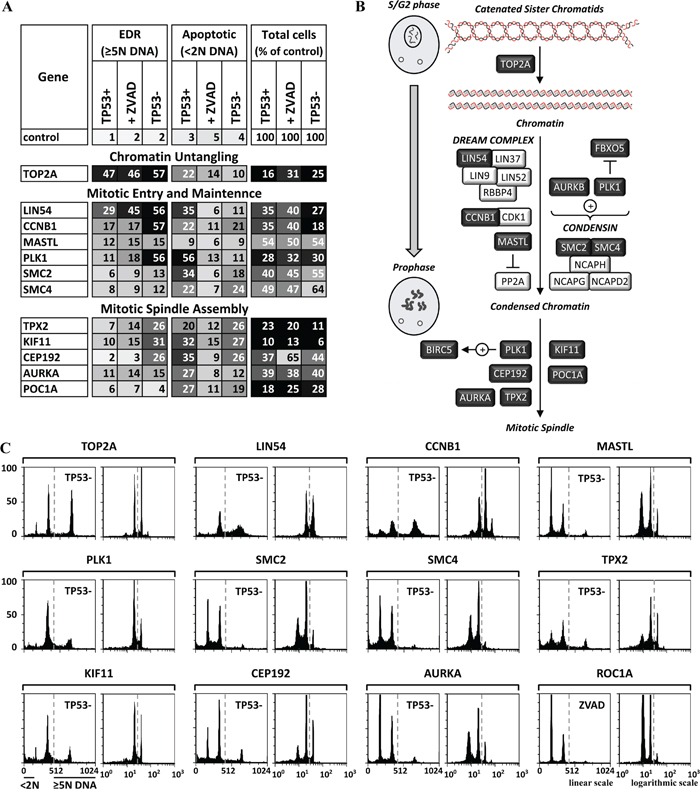
Genes essential for untangling chromatin and entrance into mitosis were also essential to prevent EDR **A.** Twelve of the validated genes have functions that untangle chromatin from G2 phase until metaphase, drive cells from G2 phase into mitotic prophase, and assemble the mitotic spindle during the prophase to prometaphase transition (illustrated in **B.** dark boxes, light font). The results of the three validation assays (TP53+ cells, TP53+ cells +ZVAD, and TP53- cells) carried out on each gene are summarized in panel A, and the FACS profiles are in Supplementary Figure S2B, S2C. FACS profiles for the validation assay with the strongest EDR signals are in panel **C.** The FACS profiles are consistent with induction of endoreplication or mitotic slippage when one of these genes is depleted.

**Figure 6:**
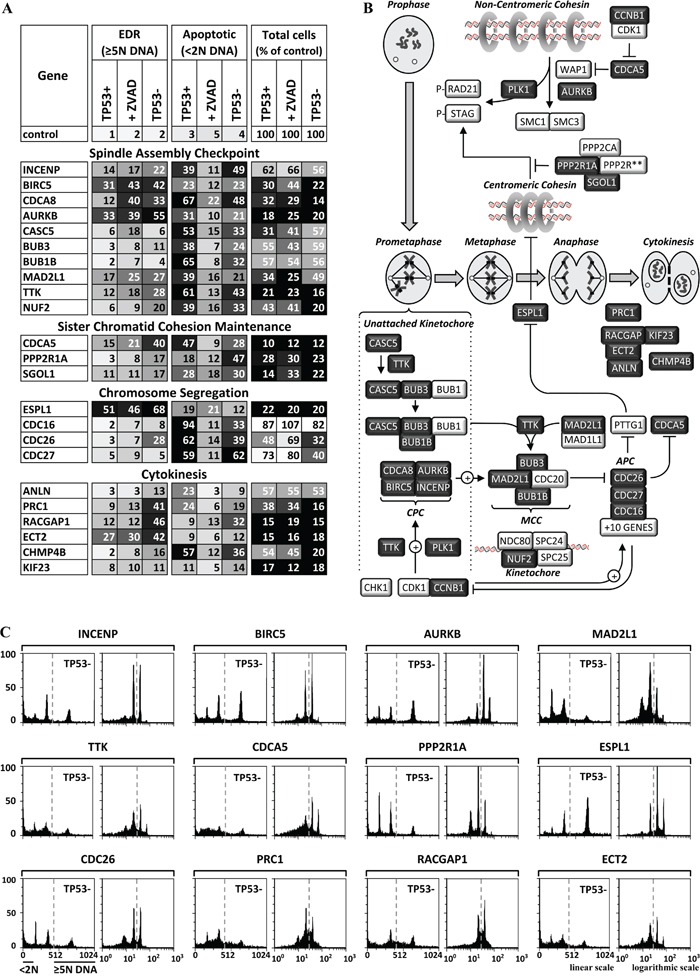
Genes essential for completion of mitosis and cytokinesis were also essential to prevent EDR **A.** Twenty-three of the validated genes have functions required for the spindle assembly checkpoint during prometaphase, maintenance of sister chromatid cohesion from S phase until metaphase, chromosome segregation during anaphase, or cytokinesis (illustrated in **B.** dark boxes, light font). The results of the three validation assays (TP53+ cells, TP53+ cells +ZVAD, and TP53- cells) carried out on each gene are summarized in panel A, and the FACS profiles are in Supplementary Figure S2D, S2E. FACS profiles for the validation assay with the strongest EDR signal for 12 genes are in panel **C.** The FACS profiles are consistent with induction of endoreplication or mitotic slippage when one of these genes is depleted.

Since induction of EDR can induce DNA damage dependent apoptosis [29], the level of excess DNA might depend on how rapidly EDR triggers apoptosis. This hypothesis was confirmed by culturing cells in the presence of MLN4924, a small molecule that selectively inhibits cullin based ubiquitin ligases by preventing their neddylation [48]. FACS analysis (Figure 3A) revealed that accumulation of cells with ≥5N DNA (EDR signal) was followed rapidly by accumulation of cells with <2N DNA (apoptotic cells). Moreover, accumulation of apoptotic cells was accompanied by disappearance of EDR cells (Figure 3B). To determine whether or not apoptosis was linked directly to EDR, apoptosis was inhibited by addition of Z-VAD(OMe)-FMK (ZVAD), an irreversible inhibitor of caspases. As expected, ZVAD inhibited apoptosis, and increased the fraction cells with excess DNA by 2.5-fold (Figure 3C, 3D). Therefore, validation of candidate genes was carried out in the presence and absence of ZVAD (TP53+ cells ±ZVAD; Figures 4A, 5A, 6A). ZVAD reduced the fraction of apoptotic signal with on average 23% (Supplementary Figure S3). With three exceptions (ESPL1, RBX1 and DTL), ZVAD increased the level of excess DNA an additional 5% to 10% relative to TP53+ cells alone.

### EDR induced apoptosis reduced cell viability

The tables in Figures 4A, 5A and 6A include the total number of cells remaining in each validation assay, as well as the extent of EDR and apoptosis induced by the siRNA. The reduction in the number of cells remaining, relative to controls, revealed the efficacy of each siRNA tested under different experimental conditions. Reduced numbers of cells meant that the siRNA had depleted a gene required for cell proliferation. The fact that at least three independent siRNAs for each validated gene reduced the number of cells to a similar extent confirmed the specificity of the observed effects. Furthermore, some siRNAs exhibited different effects on either EDR or apoptosis when assayed either in the presence of ZVAD or in the absence of TP53. Nevertheless, they inhibited cell proliferation to similar extents, thereby confirming that the effects of ZVAD and TP53 on EDR and apoptosis were not experimental artifacts. For example, siRNA depletion of PLK1 in three different validation assays reduced cell proliferation to 30% of control, but the extent of EDR varied from 11% to 56%. Similar comparisons were made for CDCA5, RACGAP1 and TPX2. The fraction of cells with normal DNA content (2N-4N) revealed that induction of EDR induced apoptosis that resulted in reduced viability (Supplementary Figure S3).

### Genes that prevent EDR restrict genome duplication to once per cell division

Of the 85 genes subjected to three validation assays, only 42 were validated as essential to prevent EDR (Table 1). These genes induced an EDR signal in FACS profiles (Supplementary Figure S2) that was at least 5-fold above control cells in one or more assays (Supplementary Figure S3). Twenty-nine genes were validated in TP53+ cells. Addition of ZVAD revealed 11 additional genes. Assays in TP53- cells revealed an additional two genes. For example, CASC5 depletion induced EDR was detected only when apoptosis was inhibited, whereas CDP192 depletion induced EDR only in the absence of TP53 (Figure 3E). The validation assays with the most robust signal are presented in Figures 4, 5, and 6, and all the FACS profiles are presented in Supplementary Figure S2.

**Table 1:** ‘This study’ identified and validated 42 genes that are essential for preventing excess DNA replication in HCT116 cells

Genes essential to prevent EDR			
Name	New	Prevents in mice	
		Aneuploidy/Polyploidy	Tumorigenesis
**Origin Licensing Inhibition**			
FBXO5/Emi1			
GMNN/Geminin			
CUL1/Cullin 1			
NEDD8			
RBX1/ROC1			
DTL/Cdt2/DCAF2			
DDB1			
**Chromatin Untangling**			
TOP2A/Topoisomerase II alpha			
**Mitotic Entry and Maintenance**			
LIN54			
CCNB1/Cyclin B1	+		
MASTL/Greatwall	+		
PLK1/Polo-like kinase 1		+	+
SMC2	+		
SMC4	+		
**Mitotic Spindle Assembly**			
TPX2		+	+
KIF11/Eg5/Kinesin-11		+	+
CEP192	+		
AURKA/Aurora kinase A		+	+
POC1A/WDR51A	+		
**Spindle Assembly Checkpoint**			
INCENP		+	
BIRC5/Survivin		+	
CDCA8/Borealin		+	
AURKB/Aurora kinase B		+	
CASC5/D40/KNL1	+		
BUB3	+	+	
BUB1B	+	+	
MAD2L1/MAD2	+	+	+
TTK/Mps1		+	+
NUF2	+		
**Sister Chromatid Cohesion**			
CDCA5/Sororin			
PPP2R1A/PP2A-alpha	+		
SGOL1/Sgo1/Shugoshin-like 1	+	+	+
**Chromosome Segregation**			
ESPL1/Separase		+	+
CDC16/APC6	+		
CDC26/APC12	+		
CDC27/APC3	+		
**Cytokinesis**			
ANLN/Anillin			
PRC1			
RACGAP1			
ECT2			
CHMP4B	+		
KIF23/MKLP1/Kinesin-23			

The first gene name is from the Human Gene Nomenclature Committee (HUGO). In some cases, other names are provided that appear in recent literature. These genes participate in one or more of eight cell cycle events (indicated). To our knowledge, 17 of these genes have not been identified previously (‘New’), whereas the remaining genes have been identified previously in high throughput screens on HeLa [38] and U2OS [39] cells, or in single gene studies using siRNAs or chemical inhibitors. Some studies reported the appearance of aneuploid or polyploid cells during mouse development (aneuploid/polyploid), and some studies reported an increased incidence of tumorigenesis when the expression level of the indicated gene was altered (‘tumorigenesis’). See Discussion, ‘Excess DNA replication is linked to aneuploidy and tumorigenesis’.

Sixteen of the genes in Table 1 were previously identified in two high throughput screens for cell cycle regulators [38, 39], and 11 other genes have been identified in single gene studies using either siRNA or a chemical inhibitor (cited below), thereby confirming that the criteria used for validation in the present study were adequate to identify all of the genes essential for preventing EDR in the cells tested. An extensive search of the literature revealed that 17 of the 42 genes (40%) in Table 1 have not previously been reported to prevent EDR. All 42 genes are components of specific cell cycle events that prevent either DNA re-replication or unscheduled endoreplication.

### Origin licensing inhibition

Nuclear DNA replication occurs only once during each cell division. When S phase begins, assembly of prereplication complexes on chromatin (‘origin licensing’) is blocked until mitosis is completed. If licensing occurs during S phase, cells accumulate with a single enlarged nucleus containing heterogeneous amounts of DNA (>4N to 8N or more) that leads to DNA damage and apoptosis. Therefore, multiple pathways exist that can inactivate the helicase loader during S phase (Figure 4B), thereby preventing both reloading of MCM helicases at activated replication origins, and licensing of new replication origins [23, 28, 49].

Four activities known to prevent origin licensing during S phase were essential to prevent DNA re-replication: GMNN, FBXO5, CRL1 AND CRL4. GMNN and FBXO5 prevented EDR that induced TP53 independent apoptosis (Figure 4A). GMNN binds to CDT1 and prevents its ability to load MCM helicases onto DNA replication origins (Figure 4B). FBXO5 inhibits the APC, an E3 ubiquitin ligase that targets both GMNN and CCNA for degradation, thereby preventing their destruction until mitosis. Since the APC is also required for the metaphase to anaphase transition (Figure 6B), inhibiting the APC induces unscheduled endoreplication.

Cullin-RING based E3 ubiquitin protein ligases (CRLs) target proteins for ubiquitin-dependent degradation. They each contain a Cullin subunit. However, of the eight Cullin genes, only CUL1 was selected by the HTS and validated, revealing the importance of CRL1 in preventing EDR. CRL1 consists of CRL1 targets the CDK-dependent phosphorylated forms of CDT1 and ORC1. Although CDK2·CCNA phosphorylation of CDT1, ORC1 and CDC6 inhibits their ability to load the MCM helicase onto replication origins and converts CDT1 and ORC1 into CRL1 substrates, neither CDK2 nor CCNA were found to be essential to prevent EDR. The reason presumably arises from redundant activities. CDK2 activity can be replaced by CDK1, and CCNA by CCNE [8]. Alternatively, since CDK2 is normally required for initiation of S phase, EDR would not occur in its absence.

CRL4 contains five components, four of which (DTL, DDB1, RBX1 and NEDD8) were essential to prevent EDR. However, neither CUL4A nor CUL4B (either of which can serve in CRL4) were selected, because their function is redundant. CRL4 targets CDT1 only when it is associated with PCNA and DNA. NEDD8 and RBX1 (the RING subunit), essential components of all CRLs, were selected and validated in all three assays, consistent with a requirement for both CRL1 and CRL4 in preventing DNA re-replication.

### Chromatin untangling

**Figure 7:**
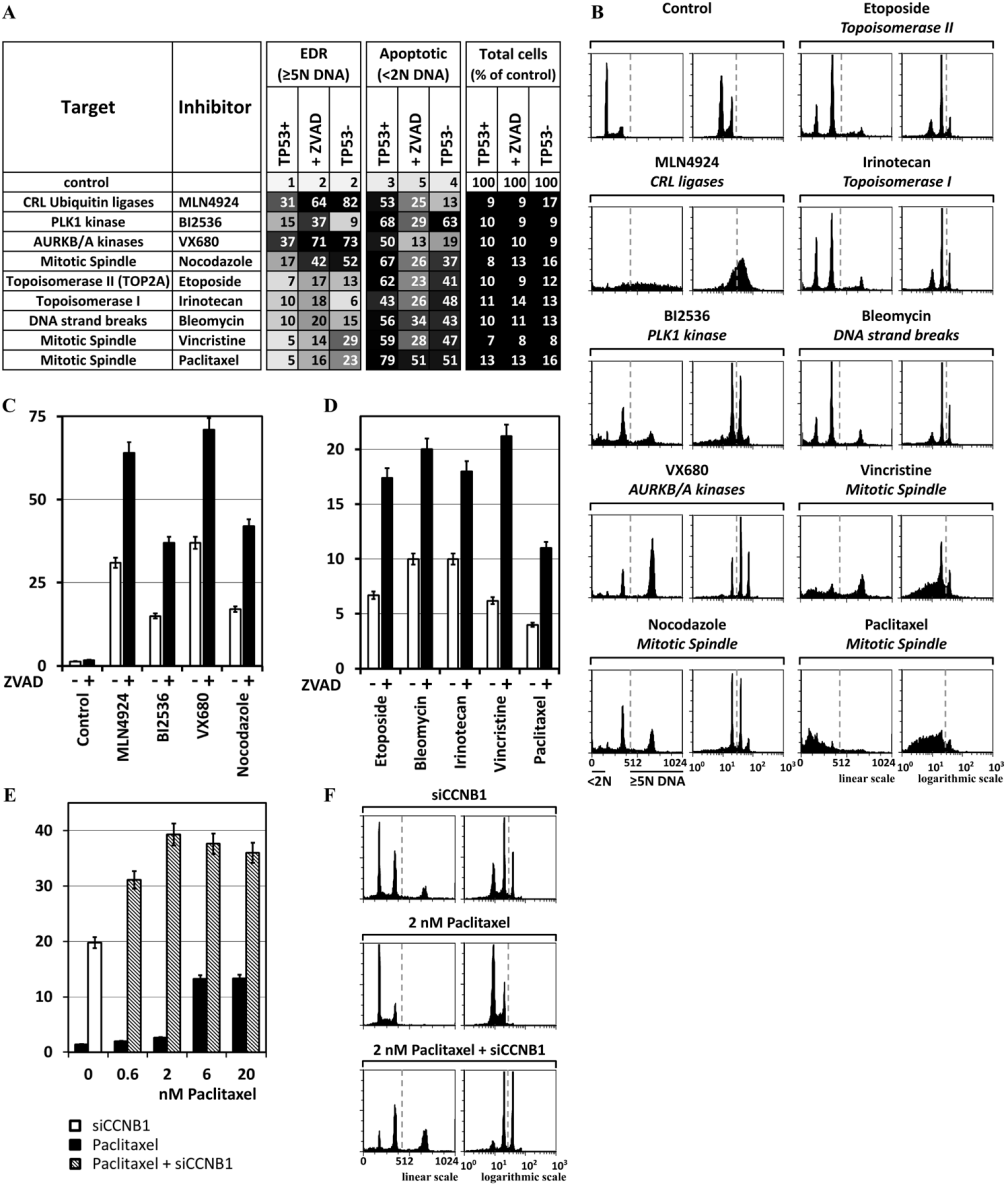
Chemical inhibitors also can induce EDR in cancer cells MLN4924 (0.2μM) inhibits neddylation of cullin based E3 ubiquitin ligases. BI2536 (25nM) inhibits PLK1. Etoposide (2μM) inhibits TOP2A. VX680 (0.6μM) inhibits AURKA and AURKB. Nocodazole (50ng/ml) binds tubulin and inhibits polymerization of microtubules. Cells were culture for 3 days in the presence of the indicated chemical, and then subjected to FACS. **A.** The results of the three validation assays (TP53+ cells, TP53+ cells +ZVAD, and TP53- cells) carried out on each chemical inhibitor are summarized. **B.** FACS profiles for the validation assays with the strongest EDR signal. **C.** Addition of 15μM ZVAD to HCT116(TP53+) cells revealed that, on average, 50% of the cells with excess DNA underwent apoptosis. **D.** HCT116(TP53+) cells were treated for 3 days with 2μM Bleomycin, 2μM Irinotecan, 60nM Vincristine, or 20nM Paclitaxel and then subjected to FACS. ZVAD increased, on average, 2.5-fold the percentage of cells with >5N DNA content. **E.** Cells were transfected with siCCNB1 and 24 later Paclitaxel was added to determine whether or not depletion of a gene essential to prevent EDR could promote the ability of Paclitaxel to induce EDR. At low Paclitaxel concentrations (2nM), the effect of siCCNB1 was synergistic. **F.** FACS profiles of selected samples. All the FACS profiles are in Supplementary Figure S2F.

Topoisomerase II untangles sister chromatids when replication forks from neighboring replicons collide and terminate replication (Figure 5B), and it continues to untangle DNA during mitosis [50]. Depletion of the enzymatic subunit (TOP2A) of topoisomerase II (Figure 5A, 5C; Supplementary Figure S2B) or inhibition by etoposide (Figure 7) induced EDR in the form of endoreplication. Therefore, TOP2A activity was essential only after DNA replication was completed. In contrast to siRNA, topoisomerase chemical inhibitors kill cells by trapping topoisomerases on DNA rather than by reducing enzymatic activity, which results in incomplete DNA replication and the appearance of aneuploid cells [51].

### Mitotic entry and maintenance

Of the genes involved in mitotic entry and maintenance, only eight were essential to prevent EDR (Figure 5A) with endoreplication type EDR (Figure 5C; Supplementary Figure S2B). The five-subunit condensin complex contributes to the assembly of condensed chromosomes and remains with the chromatids throughout mitosis [52]. siRNAs against either SMC2 or SMC4 produced a moderate induction of endoreplication (Figure 5A; Supplementary Figure S2B). LIN54, a member of the five-subunit core module of the DREAM complex (Figure 5B) that is essential for mouse development and viability [53], was also essential to prevent unscheduled endoreplication due to mitotic slippage [Figure 5A, 5C; Supplementary Figure S2B; [38]. LIN54 is required for expression of multiple proteins that are essential for mitotic entry, spindle assembly and sister chromatid cohesion [53]. CDK1·CCNB1 and PLK1 kinases phosphorylate multiple targets during initiation and maintenance of mitosis. PLK1 phosphorylates FBXO5 just before nuclear envelope breakdown, thereby targeting it for ubiquitin-dependent degradation [54]. This allows CDC20 to either activate the APC or to be sequestered by the spindle assembly checkpoint (Figure 6B). CDK1·CCNB1 promotes centrosome maturation and separation, chromosome condensation and mitotic entry after nuclear envelope breakdown [55]. siRNA against cyclin B1 (CCNB1) induced endoreplication, confirming that mitotic entry is essential to prevent endoreplication [21]. CCNB1·CDK1 prevents exit from mitosis by phosphorylating several APC subunits, including CDC20, thereby maintaining the APC in an active state (Figure 6B). The protein kinase MASTL/Greatwall, inhibits PP2A-B55 [56] to accelerate entry into mitosis and block exit from mitosis. Failure to inhibit PP2A-B55 arrests the cell cycle in G2 phase.

### Mitotic spindle assembly

The bipolar mitotic spindle consists of microtubule filaments with one end attached to the kinetochore complex located at each chromosomal centromere, and the other end attached to one of the two centrosomes in the cytoplasm. Of the genes involved in spindle assembly, only PLK1, CEP192, TPX2, AURKA, KIF11 and POC1A were essential to prevent EDR (Figure 5A, 5B). PLK1 stabilizes the initial kinetochore-microtubule attachments and promotes spindle formation by activating the chromosome passenger complex through phosphorylation of BIRC5 [57]. Consequently, PLK1 depletion induces EDR, mitotic arrest, monoastral spindle arrays, aneuploidy, and apoptosis. siPLK1 induced EDR in TP53- cells, but extensive apoptosis in TP53+ cells (Figure 5C; Supplementary Figure S2B). CEP192 is critical for centrosome biogenesis; it binds Aurora kinase A (AURKA) and PLK1, targets them to centrosomes, and promotes sequential activation of both kinases via phosphorylation [58]. TPX2 is essential for spindle assembly and chromosome segregation during prometaphase [59]. TPX2 regulates the activity of KIF11/Eg5, a kinesin that functions early in mitosis to push the spindle poles apart by pulling microtubules past one another. Suppression of KIF11 activity activates SAC, resulting in mitotic arrest [60]. TPX2 also stabilizes the active conformation of AURKA, which is required for building a bipolar spindle regulating centrosome separation and microtubule dynamics. POC1A and POC1B act together in human cells to ensure centriole integrity [61].

### Spindle assembly checkpoint

Chromosome segregation is delayed until the kinetochores on sister chromatids are firmly attached to spindle fibers from opposite poles (metaphase). The spindle assembly checkpoint (SAC) prevents chromosome segregation by preventing CDC20 from joining the APC until metaphase has been achieved [62]. In the absence of SAC activity, ESPL1/Separase cleaves centromeric cohesin, and cells exit mitosis irrespective of chromosome-spindle attachments due to ‘mitotic slippage’ [63].

SAC has four major components that involve at least 35 genes, of which 13 were required to prevent EDR (Figure 6A, 6B). CASC5 is a scaffold on which BUB1, BUB1B/BUBR1 and BUB3 assemble to form a precursor to the mitotic checkpoint complex (MCC) consisting of MAD2L1, BUB1B, BUB3 and CDC20. In the absence of complete kinetochore-microtubule attachments, the chromosome passenger complex (CPC), consisting of INCENP, BIRC5/Survivin, CDCA8/Borealin and AURKB/Aurora kinase B, promotes the recruitment of the MCC to the kinetochore in a series of events catalyzed by the TTK/Mps1 kinase [57]. CPC activity is regulated by multiple phosphorylation events that involve CDK1·CCNB1, TTK, PLK1 and CHK1. The net result is that the SAC delays cells in prometaphase by preventing CDC20 from joining the APC to form an active ubiquitin ligase, thereby promoting correct kinetochore-microtubule attachments prior to chromosome segregation. The NDC80 complex allows kinetochores to maintain load-bearing tip attachments during both microtubule assembly and disassembly [64]. All four CPC subunits, five of the eight proteins involved in MCC assembly (CASC5, TTK, BUB3, BUB1B and MAD2L1), the NUF2 subunit of the NDC80 kinetochore complex, and three of the 13 APC subunits were required to prevent EDR.

### Sister chromatid cohesion

Sister chromatids are linked together during S phase by cohesin rings. Disruption of these rings results in premature separation of sister chromatids [65]. CDCA5/Sororin maintains sister chromatid cohesion during S phase by preventing WAP1 from dissociating noncentromeric cohesin (Figure 6B). CDCA5 is phosphorylated by CDK1·CCNB1 during prophase and targeted for degradation by the APC during anaphase. Of the 14 genes involved directly in cohesin assembly, only CDCA5 was essential to prevent EDR (Figure 6A; Supplementary Figure S2C).

Non-centromeric cohesin is dissociated during prophase. PLK1 phosphorylates the cohesin subunit STAG1, and CDK1·CCNB1 phosphorylates CDCA5, which allows WAP1 to dissociate non-centromeric cohesin (Figure 6B). AURKB is also required, but its function is undefined. Centromeric cohesin is protected from phosphorylation by protein phosphatase 2A (PP2A), which is targeted to centromeres by SGOL1/Shugoshin-like. PP2A preserves centromeric cohesion until metaphase. PLK1, AURKB, ESPL1, SGOL1 and the ‘constant regulatory subunit A’ (PPP2R1A) of PP2A were essential to prevent EDR.

### Chromosome segregation

Assembly of the metaphase spindle inactivates the CPC, which results in dissociation of the MCC. This allows formation of the APC·CDC20 complex that ubiquitinates PTTG1/Securin, a specific inhibitor of the ESPL1 protease (Figure 6B). Centromeric cohesin is removed during the metaphase to anaphase transition when ESPL1 cleaves the STAG2 subunit. ESPL1 was essential to prevent endoreplication (Figure 6A, 6C). The simultaneous destruction of PTTG1 and CCNB1 elicited by APC·CDC20 links chromosome segregation to the dissolution of the SAC during mitotic exit [66].

### Cytokinesis

Cytokinesis begins with assembly of a contractile ring composed of a filamentous network of actin, myosin II, and septin [67]. Immediately following chromosome segregation, centralspindlin (KIF23, RACGAP1) links the mitotic spindle to the plasma membrane with the help of ECT2. Binding of ECT2 to RACGAP1 at the spindle midzone induces formation of the contractile ring, and an ANLN/Anillin ·ECT2 complex stabilizes its position. PRC1 regulates formation of the midzone by stimulating PLK1 phosphorylation of RACGAP1 to allow recruitment of ECT2 to the central spindle. CHMP4B, a component of the ESCRT-III complex, functions in the final stage of cytokinesis. All of these genes were essential to prevent EDR.

### Chemical inhibitors confirmed siRNA results

The proteins identified above as essential to prevent EDR were selected by reducing their cellular levels using siRNA. To determine whether or not inhibiting their activity would produce a comparable result, HCT116(TP53+) cells ±ZVAD and HCT116(TP53-) cells were challenged with specific chemical inhibitors. MLN4924 inhibits neddylation of cullin based ubiquitin ligases essential for their activation. BI2536 inhibits PLK1. VX680 inhibits AURKB. Nocodazole inhibits mitotic spindle assembly. In each case, the chemical inhibitor recapitulated the siRNAs ability to induce EDR (Figure 7A) as well as the type of EDR (Figure 7B). MLN4924, like siNEDD8, induced DNA re-replication. BI2536 and VX680, like siPLK1 and siAURKB, respectively, induced unscheduled endoreplication due to mitotic slippage. Nocodazole, a specific inhibitor of microtubule assembly, like siINCENP, siBIRC5, siAURKB and siCDCA8 (the chromosome passenger complex) induced unscheduled endoreplication due to mitotic slippage. Moreover, in each case, ZVAD increased the level of EDR from 2 to 2.5-fold (Figure 7C). Thus, chemical inhibitors specific for proteins previously identified by siRNA suppression of gene activity confirmed the importance of at least five cell cycle events in preventing EDR in cancer cells.

### Several chemotherapeutic drugs induce EDR in cancer cells

Several drugs currently used in cancer chemotherapy also induced EDR, as demonstrated by a significant increase in the fraction of cells with excess DNA when challenged in the presence of ZVAD (Figure 7A). About 70% of broadly based chemotherapeutic regiments include an inhibitor of topoisomerase II. In the presence of ZVAD, Etoposide, a specific inhibitor of topoisomerase II activity, increased the level of EDR by 2.4-fold (Figure 7D). Similar results were obtained with Bleomycin (a DNA intercalating drug that induces DNA damage), Irinotecan (a Topoisomerase I inhibitor), Vincristine (an inhibitor of microtubule polymerization), or Paclitaxel (an inhibitor of microtubule depolymerization) (Figure 7A, 7D). The significance of EDR induction by various chemotherapeutic drugs was revealed only when apoptosis was inhibited with ZVAD. However, the full extent to which cells accumulate with excess DNA is limited by two facts: ZVAD inhibition of apoptosis is incomplete, and these drugs induce extensive DNA damage leading quickly to apoptosis. Thus, induction of EDR is, to some extent, part of the mode of action for several drugs currently used in cancer chemotherapy.

### Synergistic effects between chemotherapeutic drugs and siRNA

Suppression of genes that prevent EDR should stimulate the level of EDR induced by chemotherapeutic drugs, thereby enhancing the ability of chemotherapeutic drugs to induce aneuploidy and cell death. To test this hypothesis, CCNB1, a gene essential to prevent EDR in cancer cells, was depleted with siRNA, and then the cells were treated with increasing concentrations of Paclitaxel (Figure 7E). ZVAD also was included to inhibit apoptosis. The results revealed that Paclitaxel and siCCNB1 acted synergistically to induce EDR (Figure 7F). Paclitaxel alone at 2nM induced EDR in 3% of the cells, and siCCNB1 alone induced EDR in 20% of the cells. However, combining the two treatments induced EDR in 39% of the cells. This represented a 70% increase over the sum of the two treatments alone.

## DISCUSSION

A basic concept in oncology is that the genomic instability characteristic of cancer cells permits the rise of aneuploidy and polyploidy. One source of this genomic instability is a reduction in the number or the effectiveness of pathways that prevent EDR. In an effort to relate these two concepts, we undertook the first comprehensive identification of genes that prevent EDR by screening about 95% of the human genome (21,584 genes) for genes that prevent EDR in HCT116 cells, a colon cancer cell line with a stable, near diploid, karyotype [40] that is also available as isogenic cells differing only by the presence of a functional TP53 gene [68]. The results revealed 42 genes that prevented EDR by way of eight specific cell cycle events (Table 1). Seventeen of these genes have not been demonstrated previously in this capacity. Remarkably, 14 of the 42 genes have been shown to prevent aneuploidy or polyploidy during mouse development, and eight of them to prevent tumorigenesis.

This screen identified only those genes whose function could not be replaced completely by that of another gene. This screen did not select any gene that is required for either DNA synthesis or replication, because accumulation of excess DNA requires these processes. This screen relied upon the results of three to seven independent siRNAs. Validation assays included an additional independent siRNA and specific chemical inhibitors where available. Validation assays were done on both TP53+ and TP53- cells in order to determine whether or not this tumor suppressor enhanced or diminished EDR. These results revealed that TP53 does not prevent initiation of EDR, although in some cases, the TP53 DNA damage response activity reduced the extent of EDR. Examples include CUL1, LIN54, CCNB1, PLK1, CDCA5, CDC26, PRC1 and RACGAP1 (Supplementary Figure S3). Testing siRNAs in the presence of an apoptosis inhibitor insured that the EDR FACS signal was not lost due to DNA damage induced apoptosis. These results revealed that suppressing expression of some genes not only induced EDR, but triggered extensive apoptosis, thereby significantly reducing the EDR signal. Examples include GMNN, CUL1, LIN54, BIRC5, CDCA8 and CASC5 (Supplementary Figure S3). In fact, the ability of several genes to prevent EDR was affected both by inactivation of TP53 and inhibition of apoptosis. Examples include CUL1, LIN54, BIRC5, CDCA8, TTK and CHMP4B (Supplementary Figure S3).

**Figure 8:**
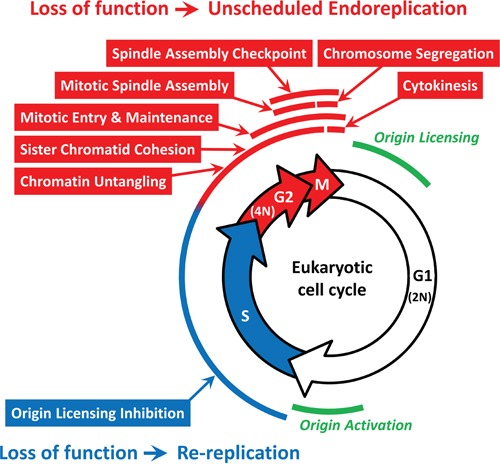
Specific cell cycle events associated with either DNA re-replication or unscheduled endoreplication The 42 genes in Table 1 participate in one or more of eight cell cycle events that restrict genome duplication to once per cell division. FACS analyses of HCT116 cells (±ZVAD) transfected with the indicated siRNA (Supplementary Figure S2) revealed that some cell cycle events (indicated in red) prevented primarily endoreplication whereas others prevented primarily DNA re-replication (indicated in blue). See text for details. Origin licensing refers to the assembly of prereplication complexes during the anaphase to G1 phase transition. Origin activation refers to the assembly of initiation complexes during the G1 to S phase transition.

An effort to determine whether or not over-expression of the putative siRNA target gene reversed the effect of protein depletion by siRNA, several genes were expressed constitutively in HCT116 cells from a retroviral vector to insure protein expression throughout the period of siRNA transfection. Unfortunately, stable clones were not recovered. This was consistent with the essential function these genes have in regulating cell cycle progression (Table 1, Figure 8).

### Excess DNA replication is linked to aneuploidy and tumorigenesis

Many of the same genes that prevent EDR in cultured cells also prevent aneuploidy or polyploidy during mammalian development or prevent tumorigenesis (Table 1). The systematic analysis of mouse models expressing haplo-insufficient and/or hypomorphic alleles of mitotic checkpoint components has revealed that reduced expression of such proteins leads to increased chromosome instability and aneuploidy. Fourteen genes that are essential to prevent EDR in cancer cells also are essential to prevent aneuploidy in mice [AURKA, BUB1B, BUB3, KIF11, MAD2L1, TPX2, TTK, SGOL1 [59, 69-78]. The fact that the reduced expression of these genes leads to aneuploidy in mice establishes a direct link between genes identified as essential to prevent EDR and genes that are essential to prevent aneuploidy *in vivo*. Ablation of the genes for ESPL1, PLK1 or any one of the four subunits of the chromosome passenger complex (INCENP, BIRC5, CDCA8, AURKB) results in aneuploidy/polyploidy during mouse development [34-37, 79, 80]. Mutations in genes essential to prevent EDR appear to contribute to the development of specific cancers. For example, ANLN and PRC1 were mutated in cells derived from a malignant melanoma [81], and RANBP2, TTK, PP2R1A, and CEP192 were mutated in cells from pancreatic cancers [82]. Moreover, PLK1, TPX2, KIF11, AURKA, MAD2L1, TTK, SGOL1, ESPL1 not only prevent aneuploidy/polyploidy, but also prevent tumorigenesis [59, 74-80]. These results suggest that all 42 genes in Table 1 are essential to prevent aneuploidy and tumorigenesis.

### DNA re-replication versus unscheduled endoreplication

All of the 42 genes in Table 1 participate in eight specific cell cycle events that occur at different times during the mammalian cell cycle (Figure 8). Therefore, each of these events plays an essential role in restricting genome duplication to once per cell division. In principle, genes that are essential to prevent EDR prevent either DNA re-replication or unscheduled endoreplication, depending on whether the gene's function is required during S phase or after S phase, respectively. Endoreplication produces polyploid cells that result from one or more rounds of genome duplication in the absence of an intervening mitosis or cytokinesis. These cells contain a single nucleus with an integral multiple of 4N DNA. Consequently, they produce a distinctive FACS signature with peaks at 8N, 16N and 32N DNA content. DNA re-replication, however, produces aneuploid cells that result from incomplete genome duplication. These cells contain a single nucleus with a heterogeneous DNA content between 4N and 8N. Consequently, their FACS signature is indistinguishable from aneuploid cells that result either from apoptotic polyploid cells, or from incomplete DNA replication during the S phase following mitotic slippage. Therefore, the FACS profiles of HCT116 cells (±ZVAD) transfected with a gene selective siRNA were sorted into those with and those without a strong, easily recognized, 8N peak. The results clearly revealed DNA re-replication occurred predominantly, if not exclusively, when the block to origin licensing during S phase was disrupted, and unscheduled endoreplication occurred predominantly, if not exclusively, when cells were prevented from entering and completing mitosis and cytokinesis (Figure 8).

Genes required for sister chromatid cohesion (CDCA5, SGOL1, PPP2R1A) or chromatin untangling (TOP2A) are active throughout S, G2 and early M phases, but they were not essential to prevent EDR until cells attempted to transit mitosis, and therefore their absence produced a strong endoreplication signal (Supplementary Figure S2B, S2C). In the case of TOP2A, selective inhibition by Etoposide, which causes DNA strand breaks, induced both endoreplication and DNA re-replication signatures (Supplementary Figure S2F), consistent with a requirement during S phase as well as G2 and M phases.

Other genes, such as those required for mitotic entry and maintenance (CCNB1, MASTL, PLK1, SMC2, SMC4), mitotic spindle assembly (KIF11, TPX2, AURKA, POC1A), spindle assembly checkpoint (AURKB, BIRC5, CDCA8, INCENP, CASC5, TTK, MAD2L1, BUB3, BUB1B), and chromosome segregation (ESPL1) produced a distinct population of cells with 8N and sometimes 16N DNA content. Since these profiles mimicked the effects of antimitotic drugs such as Nocodazole, and arrested cell division after completion of S phase, endoreplication was a consequence of mitotic slippage. Chemical inhibition of aurora kinases A and B (VX680) or PLK1 (BI2536) induced endoreplication to a much greater extent than did siRNAs against these genes, thereby confirming that their role in preventing endoreplication. Similarly, genes required for cytokinesis (ECT2, PRC1, RACGAP, CHMP4B, KIF23) also induce endoreplication, presumably by a mechanism analogous to mitotic slippage.

Genes that prevented origin licensing (GMNN, FBXO5, CUL1, and NEDD8) during S phase were essential to prevent DNA re-replication (Figure 4). FACS profiles in which one of these genes was inhibited revealed accumulation of cells with a continuous range of ploidy from 4N to 8N or greater as a result of incomplete DNA replication and the accumulation of double strand DNA breaks [29, 30]. Moreover, chemical inhibition of neddylation with MLN4924 (Figures 3A; Supplementary Figure S2F), which inhibits all Cullin-dependent ubiquitin ligases, resulted in a strong DNA re-replication signal, thereby confirming the effect of siNEDD8 and the requirement for both CRL1 and CRL4 in preventing origin licensing during S phase. Surprisingly, LIN54, a protein that binds directly to the CHR element in promoters of genes, such as CDK1, and whose depletion results in cytokinesis and mitotic defects exhibited both a strong DNA re-replication and a strong endoreplication FACS signature, suggesting that LIN54 regulates expression of genes required to prevent origin licensing during S phase, as well as genes required for mitosis.

Heterotypic multi-subunit complexes exemplified the fact that not all proteins within a single cell cycle event are essential to prevent EDR. Whereas the chromosome passenger complex required all four of its subunits to prevent EDR (Figure 6B), other complexes did not. Examples include the cullin-dependent ubiquitin ligases (Figure 4B), the Dream complex, Cdk1·CcnB1, and Condensin (Figure 5B), and the mitotic checkpoint complex and anaphase-promoting complex (Figure 6B). These differences revealed which subunits are both essential for activity and have a short enough half-life that their levels can be reduced effectively by a siRNA. Other subunits in the complex might be sensitive to specific chemical inhibitors. For example, CcnB1·Cdk1 is essential for both entry and maintenance of mitosis, but only CcnB1 was identified by siRNA. However, selective chemical inhibition of Cdk1 activity also results in EDR [19-22]. In some cases, suppression of protein levels by siRNA might be insufficient to induce the phenotype. In other cases, some proteins might not be essential for the activity of a multi-subunit complex, or they might be replaced by another protein with similar activity. Thus, emphasis should be placed on identification of specific events or pathways rather than on specific genes.

### Therapeutic potential

The effectiveness of chemotherapy drugs currently used against cancer could be due, at least in part, to induction of EDR-dependent apoptosis. For example, drugs that inhibit Topoisomerase I, Topoisomerase II, tubulin polymerization, or tubulin depolymerization, and drugs that induce DNA strand breaks also induced EDR. This was particularly evident when ZVAD was used to inhibit apoptosis. Furthermore, depletion of CCNB1, a gene essential for preventing EDR, increased the EDR-inducing effect of Paclitaxel at low concentrations. Thus, inhibition of a gene that is essential to prevent EDR in the presence of an established chemotherapeutic drug, such as Paclitaxel, has the potential of creating a ‘synthetic lethal’, inhibiting two or more proteins that leads to cell death under conditions where inhibition of neither protein has the same effect.

The presence or absence of checkpoint control genes and proapoptotic genes, such as TP53, can dramatically effect affect induction of EDR (e.g. siPLK1), as can the efficiency at which EDR induces apoptosis. The true extent of EDR was observed only when apoptosis was impaired. In the presence of ZVAD, the EDR signal increased significantly above control levels for 26% of the validated genes. Similarly, comparing the effects of the same siRNA on isogenic cells that differ only in TP53 revealed clearly the essential role TP53 has in EDR elimination. In the absence of TP53 activity [e.g. HCT116(TP53-) cells], more extensive EDR was observed. One notable exception was the apparent effect of TP53 on CRL4 activity. Depletion of NEDD8, DTL or DDB1 produced higher levels of EDR in the presence of TP53, suggesting that TP53 promotes EDR when CRL4 activity is suppressed.

Simply knowing which genes are essential for preventing EDR reveals the pathways that are modified in cancer cells to promote genomic instability and its associated aneuploidy and consequently allow cancer cells to evolve so as to evade anti-cancer treatments.

## MATERIALS AND METHODS

### siRNA screen

A high throughput screen (HTS) that was originally developed to identify small molecules that induced accumulation of excess DNA in cancer cells [83] was adapted to screen the Ambion ‘Silencer Select Human Genome siRNA Library V4′ for genes that are essential to prevent EDR in human cells [43]. This library contained three unique non-overlapping siRNAs for each of 21,584 genes, which represented about 95% of protein coding genes (Ensemble, GRCh37.p13) [84]. Each siRNA was transfected individually into HCT116 colorectal cancer cells, which were then cultured for three days before their DNA was stained with Hoechst 33342. Fluorescence emitted from the nucleus of individual cells attached to the surface of the plate was quantified. Isogenic HCT116(TP53+) and HCT116(TP53-) cells were provided by their originator, Dr. Bert Vogelstein, and cultured as described [68]. The cell lines were passed for fewer than 3 months following recovery from the original aliquots and were regularly authenticated by Western blots analysis including their p53 status, growth, and morphology observation.

### siRNA transfection

Cells were seeded in 6-well dishes at 0.7×10^5^ cells per well to insure they were undergoing exponential proliferation until collection. Cells were transfected with a single siRNA (40nM) at one and two days post-seeding using RNAiMAX per manufacturer's instructions. During each medium replacement, floating cells were collected and added back to the wells in order to include detached apoptotic cells in the analysis. Cells were harvested by trypsinization three days after the first transfection and combined with detached cells.

### Fluorescence activated cell sorting (FACS)

Cells were washed in phosphate buffered saline, and then 2×10^5^ cells were stained with propidium iodide and subjected to FACS analysis [46] using a FACSCalibur flow cytometer (Becton Dickinson, Mountain View, CA) according to the manufacturer instructions. Special attention was given to insuring that the cells were well separated before FACS, and that cells with 2N DNA content were coincident from one analysis to the next. Data were analyzed using FCS Express (De Novo Software).

### Chemical inhibitors

MLN4924, BI2536, Etoposide, VX680, Nocodazole, Paclitaxel, and Z-VAD(OMe)-FMK (ZVAD) were purchased from Cayman Chemical Company, USA.

### Western immuno-blotting

Equal numbers of cells were collected, lysed in Laemmli sample buffer, and subjected to electrophoresis and protein immuno-blotting. Filters were probed with specific antibodies and the signals visualized with enhanced chemiluminescence (ThermoScientific).

## SUPPLEMENTARY FIGURES


