# Supplementary material for: Identification of genes that are essential to restrict genome duplication to once per cell division
Source: Oncotarget. 2016 Apr 26;7(23):34956–76. doi: 10.18632/oncotarget.9008 (PMC5085202; doi:10.18632/oncotarget.9008)
Supplement: Supplementary file 1 [file oncotarget-07-34956-s001.pdf]

SUPPLEMENTARY FIGURES

A

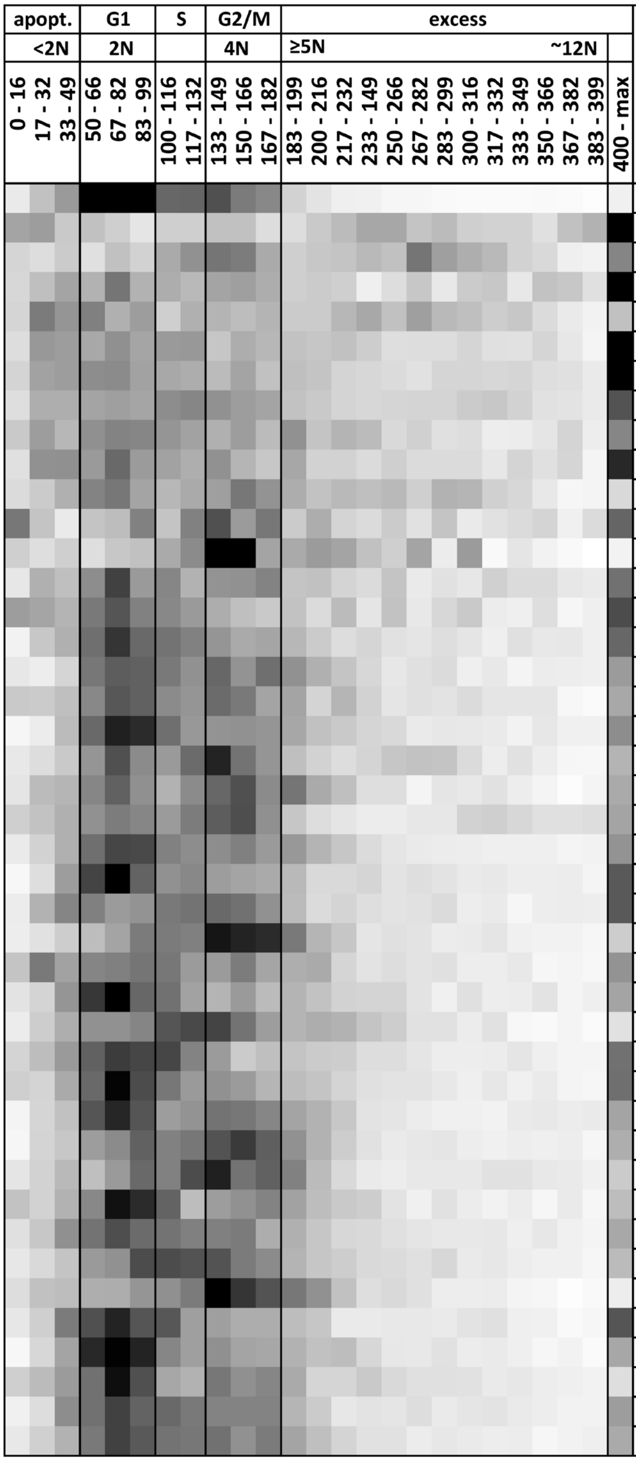

Cell count	% <2N DNA	% G1 phase	% S phase	% G2/M	% ≥5N DNA	Gene
5074	11	39	8	17	11	control
375	14	8	6	10	62	FBXO5
513	8	8	11	20	53	TPX2
419	12	17	9	16	47	INCENP
765	16	17	8	13	47	GMNN
962	14	17	12	12	46	BIRC5
1606	14	18	10	13	45	ESPL1
1800	11	16	13	17	43	TOP2A
569	13	20	11	14	42	CDCA5
959	14	20	10	14	42	CDCA8
1104	10	20	9	19	42	CUL1
228	12	14	11	22	40	PLK1
245	8	9	6	33	39	KIF11
869	10	22	11	19	38	ANLN
500	15	23	12	12	37	AURKB
1577	9	26	15	16	34	PRC1
721	5	25	14	22	34	LOC728689
773	10	24	13	21	33	CASC5
2876	6	30	14	18	33	PPP2R1A
1329	7	22	14	25	33	NEDD8
552	10	22	11	24	33	CHMP2A
2873	11	20	13	24	32	LIN54
3381	9	27	13	19	32	SGOL1
2177	8	31	13	16	32	BBC3
1300	13	19	15	22	32	FLJ40448
2398	6	17	14	33	31	LIN9
1154	16	22	13	18	31	KIF23
895	10	31	13	14	31	ARD1A
1136	9	19	19	23	31	METT11D1
1475	12	28	17	13	31	WDR78
993	10	29	13	16	31	KIAA1383
1825	7	29	12	22	31	LIN37
3590	7	21	15	28	30	CDC26
555	8	18	16	27	30	CDC27
772	11	29	12	19	30	CEP192
2702	12	25	15	19	30	BUB3
1898	7	21	19	23	30	RBX1
854	10	16	14	32	30	DTL
786	12	29	14	15	30	MSRB2
1305	8	34	12	17	30	CRKL
1420	13	29	5	21	29	CCNB1
1746	9	26	16	21	29	WDR51A
2179	9	26	15	21	29	BUB1B

(*Continued*)

B

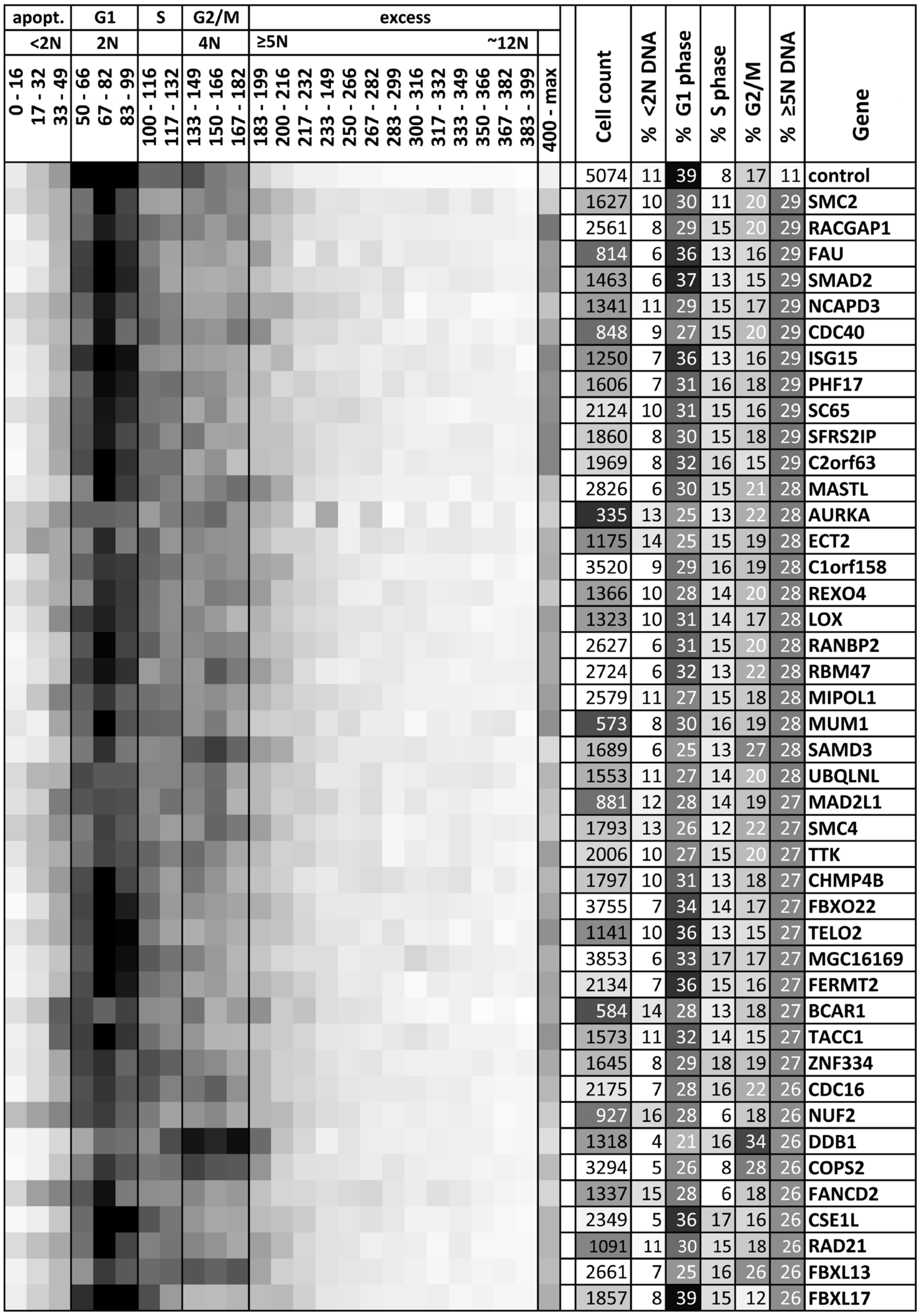

Cell count	% <2N DNA	% G1 phase	% S phase	% G2/M	% ≥5N DNA	Gene
5074	11	39	8	17	11	control
1627	10	30	11	20	29	SMC2
2561	8	29	15	20	29	RACGAP1
814	6	36	13	16	29	FAU
1463	6	37	13	15	29	SMAD2
1341	11	29	15	17	29	NCAPD3
848	9	27	15	20	29	CDC40
1250	7	36	13	16	29	ISG15
1606	7	31	16	18	29	PHF17
2124	10	31	15	16	29	SC65
1860	8	30	15	18	29	SFRS2IP
1969	8	32	16	15	29	C2orf63
2826	6	30	15	21	28	MASTL
335	13	25	13	22	28	AURKA
1175	14	25	15	19	28	ECT2
3520	9	29	16	19	28	C1orf158
1366	10	28	14	20	28	REXO4
1323	10	31	14	17	28	LOX
2627	6	31	15	20	28	RANBP2
2724	6	32	13	22	28	RBM47
2579	11	27	15	18	28	MIPOL1
573	8	30	16	19	28	MUM1
1689	6	25	13	27	28	SAMD3
1553	11	27	14	20	28	UBQLNL
881	12	28	14	19	27	MAD2L1
1793	13	26	12	22	27	SMC4
2006	10	27	15	20	27	TTK
1797	10	31	13	18	27	CHMP4B
3755	7	34	14	17	27	FBXO22
1141	10	36	13	15	27	TELO2
3853	6	33	17	17	27	MGC16169
2134	7	36	15	16	27	FERMT2
584	14	28	13	18	27	BCAR1
1573	11	32	14	15	27	TACC1
1645	8	29	18	19	27	ZNF334
2175	7	28	16	22	26	CDC16
927	16	28	6	18	26	NUF2
1318	4	21	16	34	26	DDB1
3294	5	26	8	28	26	COPS2
1337	15	28	6	18	26	FANCD2
2349	5	36	17	16	26	CSE1L
1091	11	30	15	18	26	RAD21
2661	7	25	16	26	26	FBXL13
1857	8	39	15	12	26	FBXL17

Supplementary Figure S1: DNA content analysis results for 85 genes selected for validation. Each histogram of the DNA content per cell in each well was extrapolated to represent 1000 cells. The fluorescence intensities were distributed into 25 intervals that covered the entire range of detected fluorescence. The results for each siRNA were then plotted as heat-maps in which the intensity of the color was proportional to the number of cells in the interval. In some cases, the position of nuclei with 2N DNA content (G1 phase cells) differed from the controls in the same plate. Differences in the amount of fluorescence per DNA unit that causes shifts in the DNA peaks positions of cell populations are routinely corrected during FACS by adjusting the laser PMT voltage of the instrument until the positions of the 2N or the 4N peak are the same for each sample (1). These data allowed calculation of fraction of cells with nuclear DNA content equivalent to G1 phase (2N), S phase (>2N<4N), G2/M phase (4N), apoptosis (<2N), or EDR (>5N). Gene names are from the Human Gene Nomenclature Committee (HUGO).

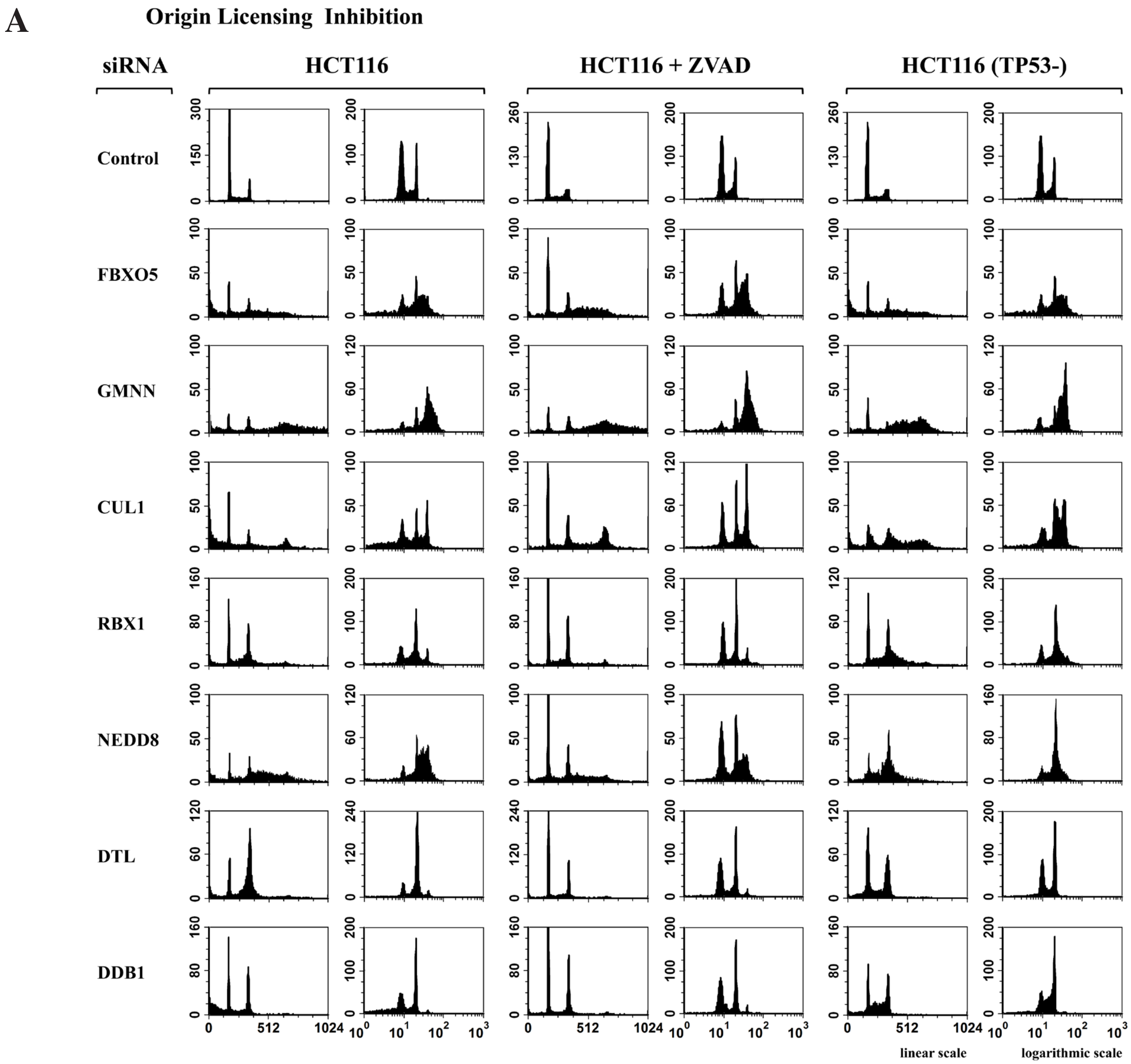
A
Origin Licensing Inhibition
siRNA
HCT116
HCT116 + ZVAD
HCT116 (TP53-)
Control
FBXO5
GMNN
CUL1
RBX1
NEDD8
DTL
DDB1
linear scale
logarithmic scale

(Continued)

B Chromatin Untangling, and Mitotic Entry and Maintenance

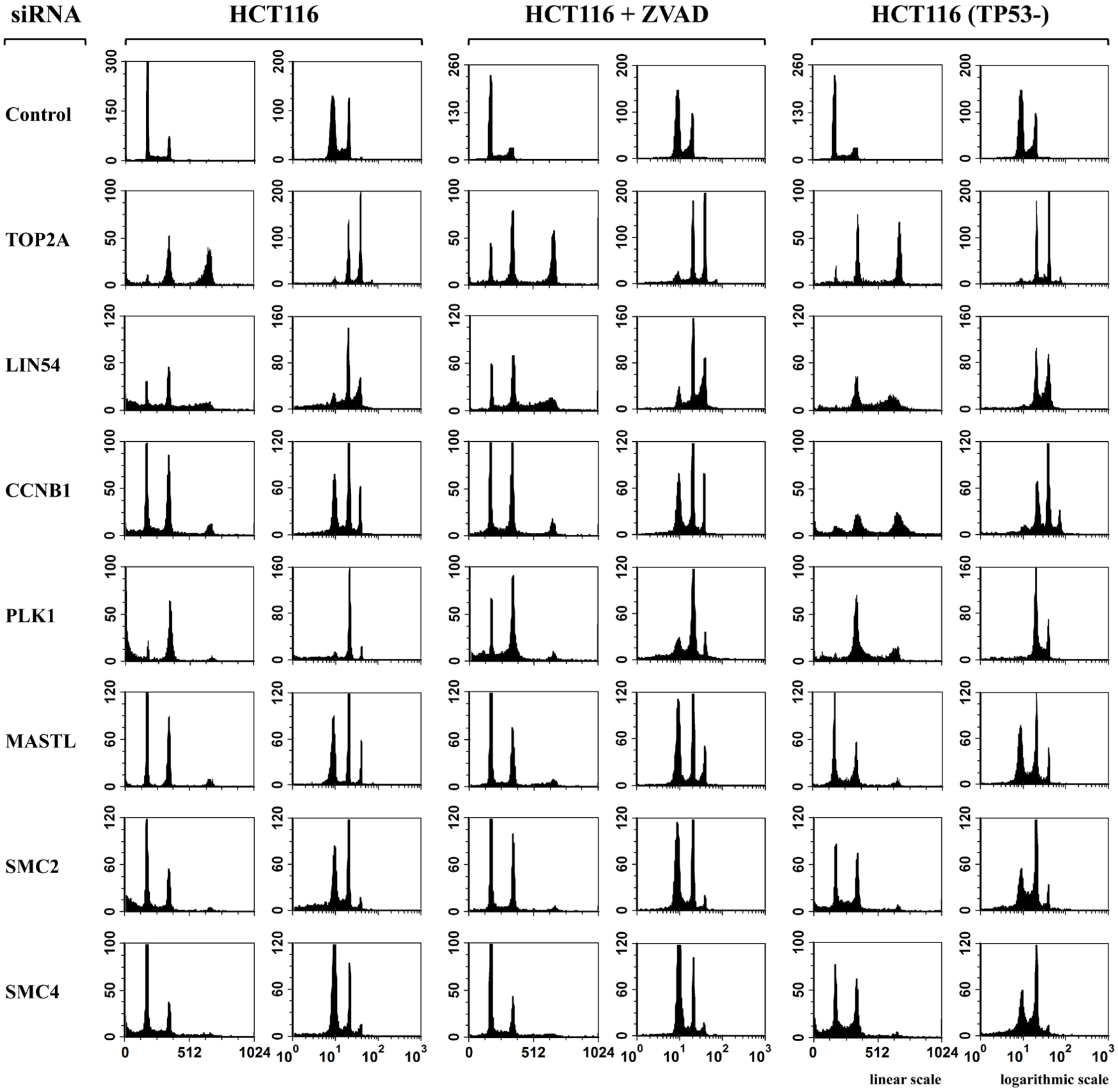

(*Continued*)

C Mitotic Spindle Assembly, Sister Chromatid Cohesion Maintenance

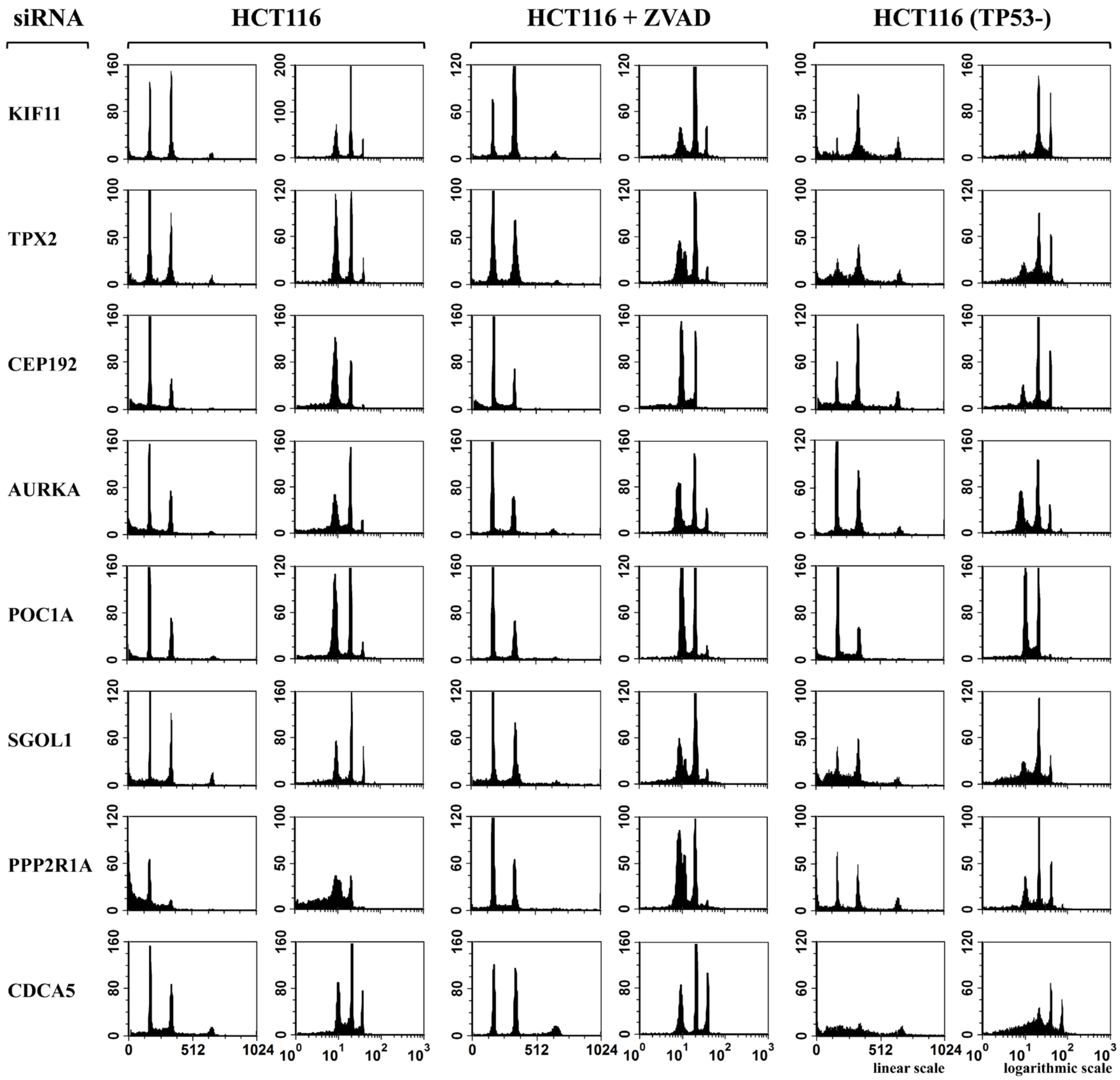

(*Continued*)

D **Spindle Assembly Checkpoint**

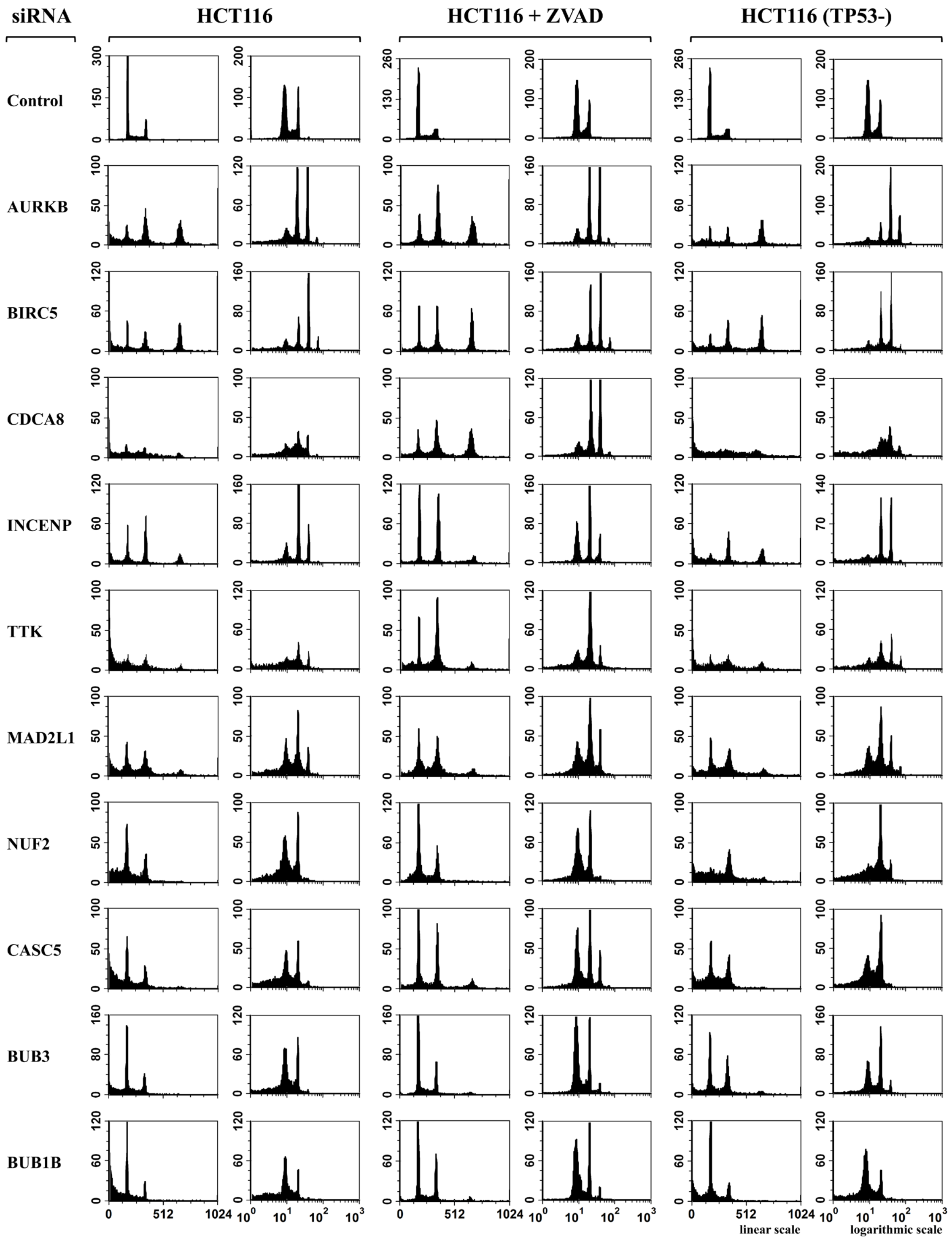

(*Continued*)

E **Chromosome Segregation, Cytokinesis**

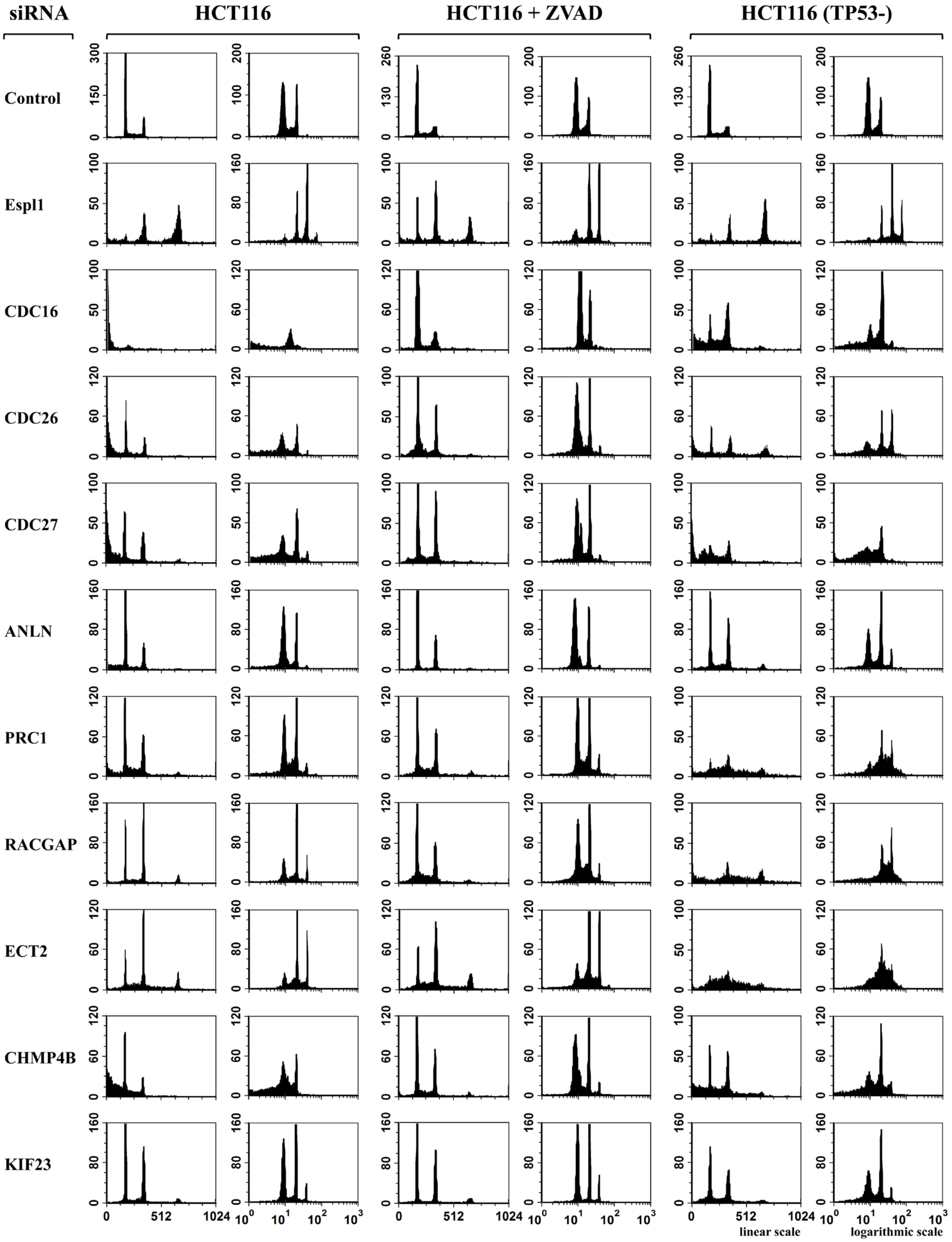

(*Continued*)

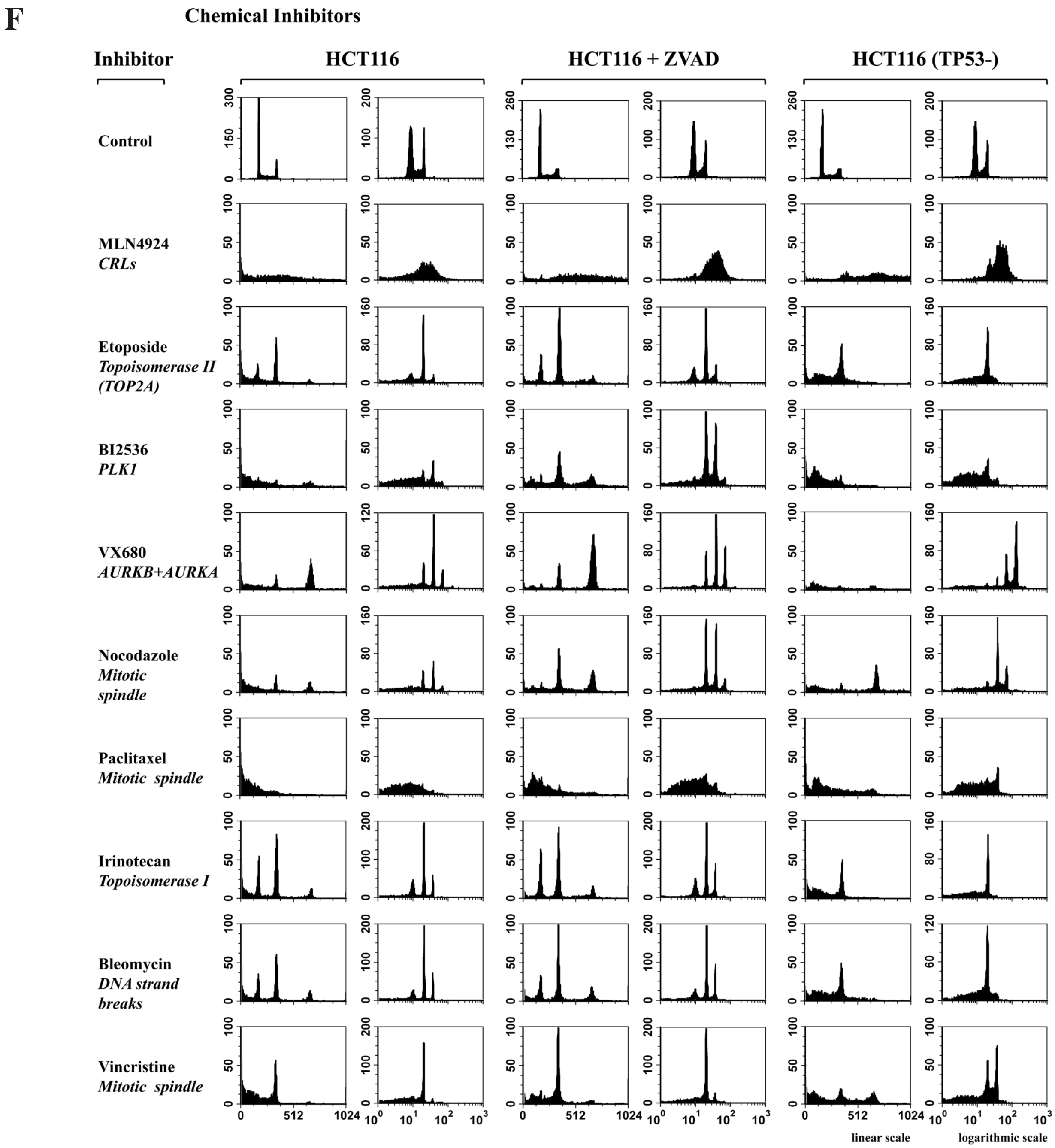
F
Chemical Inhibitors
Inhibitor
HCT116
HCT116 + ZVAD
HCT116 (TP53-)
Control
MLN4924
CRLs
Etoposide
Topoisomerase II
(TOP2A)
BI2536
PLK1
VX680
AURKB+AURKA
Nocodazole
Mitotic
spindle
Paclitaxel
Mitotic spindle
Irinotecan
Topoisomerase I
Bleomycin
DNA strand
breaks
Vincristine
Mitotic spindle
linear scale
logarithmic scale

(*Continued*)

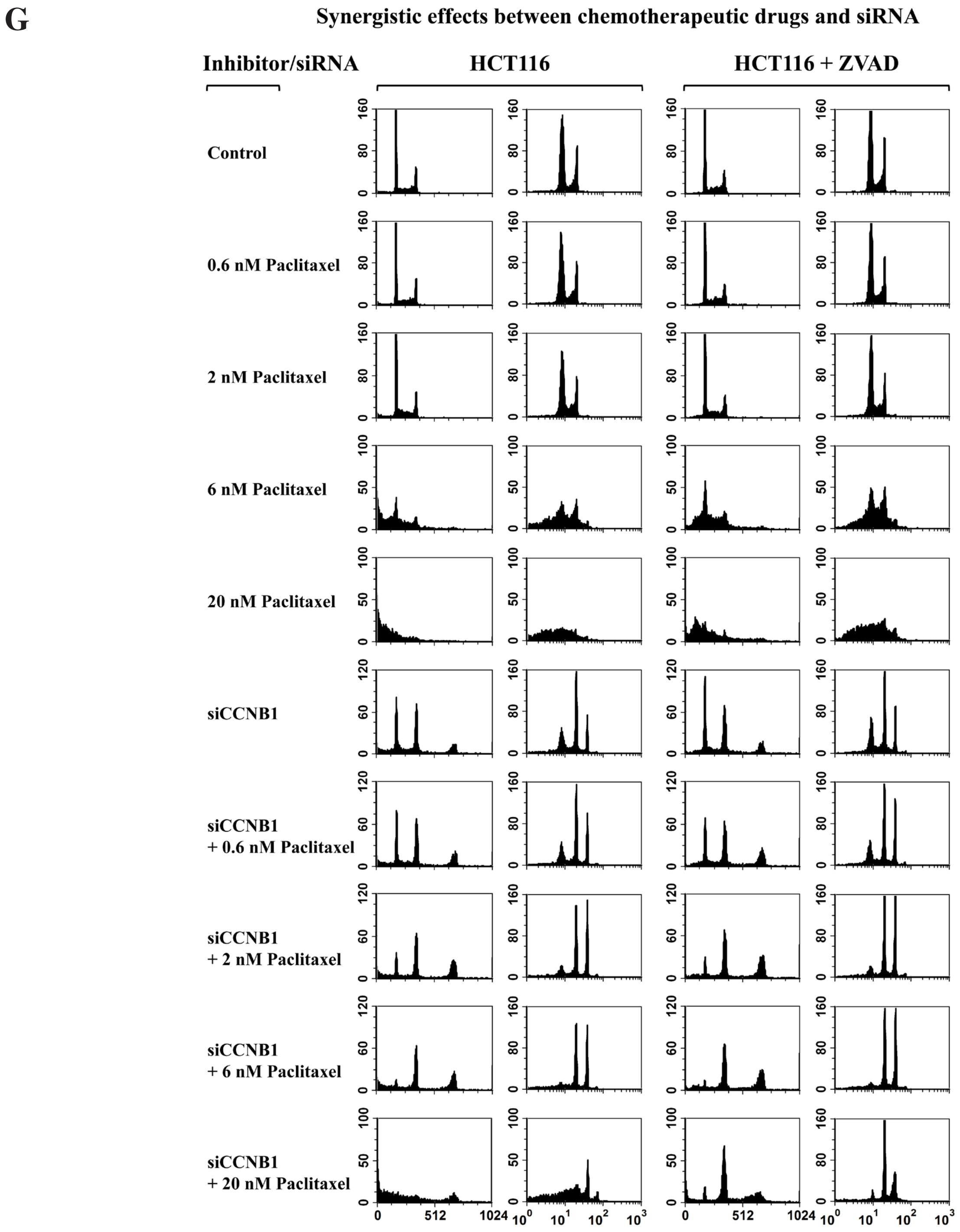

Supplementary Figure S2: Validation FACS profiles of all genes confirmed as essential for prevention of EDR. The results of the three validation assays (TP53+ cells, TP53+ cells +ZVAD, and TP53- cells) carried out on each validation siRNA are included. The genes are arranged according to their known function during the cell cycle.

Gene	HTS siRNAs	HTS % >5N DNA	HCT116(TP53+) (-)ZVAD ≥5N DNA	HCT116(TP53+) (-)ZVAD <2N DNA	HCT116(TP53+) (-)ZVAD abnormal	HCT116(TP53+) (+)ZVAD ≥5N DNA	HCT116(TP53+) (+)ZVAD <2N DNA	HCT116(TP53+) (+)ZVAD abnormal	HCT116 (TP53-) ≥5N DNA	HCT116 (TP53-) <2N DNA	HCT116 (TP53-) abnormal	Total cells (% of control) TP53+	Total cells (% of control) +ZVAD	Total cells (% of control) TP53-	2N-4N cells (% of control) TP53+	2N-4N cells (% of control) +ZVAD	2N-4N cells (% of control) TP53-	effective siRNAs	ZVAD effect Δ EDR	ZVAD effect Δ Apoposis	Δ TP53 effect Δ EDR	Δ TP53 effect Δ Apoposis
control		11	1.3	3.4	4.7	1.5	5.3	6.8	1.9	3.8	5.7	100	100	100	95	93	94		0	2	1	0
**Origin Licensing Inhibition**																						
FBXO5	7/7(5)	62	38	50	88	46	21	67	44	36	80	13	18	10	2	6	2	8	8	-29	6	-14
GMNN	7/7(5)	47	59	26	85	73	11	84	62	20	81	21	23	23	3	4	4	8	15	-16	3	-7
CUL1	3/7(5)	42	23	52	75	46	13	59	46	23	68	39	35	12	10	14	4	4	23	-40	23	-30
NEDD8	4/7(5)	33	56	17	73	57	10	67	29	11	40	17	19	32	5	6	19	5	1	-8	-27	-7
RBX1	5/7(5)	30	26	6	32	19	10	28	35	7	42	40	19	33	27	14	19	6	-8	4	9	1
DTL	2/3(3)	29	17	14	32	10	9	19	6	14	20	44	42	50	30	34	40	3	-8	-5	-11	0
DDB1	2/3(3)	26	8	22	30	11	10	21	7	20	26	35	31	63	25	24	47	3	3	-12	-1	-3
**Chromatin Untangling**																						
TOP2A	7/7(5)	43	47	22	68	46	14	60	57	10	66	16	31	25	5	12	8	8	-1	-8	10	-12
**Mitotic Entry and Maintenance**																						
LIN54	2/3(3)	32	29	35	63	45	6	51	56	11	68	35	40	27	13	20	9	3	16	-29	27	-24
CCNB1	4/7(5)	29	17	22	38	17	11	28	57	21	78	35	40	18	22	29	4	5	1	-11	40	-1
MASTL	2/3(3)	28	12	9	21	15	6	22	15	9	24	54	50	54	43	39	41	3	3	-3	3	0
PLK1	7/7(5)	40	11	56	67	18	13	31	56	11	68	28	32	30	9	22	10	8	7	-43	45	-44
SMC2	3/7(5)	29	6	34	40	9	6	15	13	18	31	40	45	55	24	38	38	4	2	-28	6	-16
SMC4	3/7(3)	27	8	22	30	9	7	15	12	24	36	49	47	64	34	40	41	4	1	-15	4	2
**Mitotic Spindle Assembly**																						
TPX2	5/7(5)	53	7	20	27	14	12	26	26	26	53	23	20	11	17	15	5	6	7	-8	19	6
KIF11	7/7(5)	39	10	32	42	15	15	30	31	27	58	10	13	6	6	9	3	8	5	-17	22	-6
CEP192	2/3(3)	30	2	35	37	3	9	12	26	26	52	37	65	44	23	57	21	3	1	-26	24	-9
AURKA	5/7(5)	29	11	27	38	14	8	22	15	12	27	39	38	40	24	30	29	6	2	-18	4	-15
POC1A	3/7(3)	29	6	27	33	7	11	17	4	19	23	18	25	28	12	21	22	4	0	-16	-2	-8
**Spindle Assembly Checkpoint**																						
INCENP	7/7(5)	47	14	39	53	17	11	28	22	49	71	62	66	56	29	48	16	8	3	-28	9	10
BIRC5	4/7(5)	46	31	23	54	43	12	55	42	23	65	30	44	22	14	20	8	5	12	-11	11	-1
CDCA8	5/7(5)	42	12	67	79	40	22	62	33	48	81	32	29	14	7	11	3	6	28	-45	22	-20
AURKB	7/7(5)	37	33	31	64	39	10	49	55	21	76	18	25	20	6	13	5	8	6	-21	22	-10
CASC5	4/7(5)	33	6	53	60	18	15	32	6	33	39	31	41	57	13	28	35	5	12	-39	0	-21
BUB3	2/3(3)	30	3	38	41	8	7	14	11	24	36	55	43	59	32	37	38	3	4	-31	8	-13
BUB1B	2/3(3)	29	2	65	67	7	8	15	4	32	36	57	54	56	19	46	36	3	5	-57	2	-33
MAD2L1	2/3(3)	27	17	39	55	25	16	41	27	21	48	34	25	49	15	15	26	3	9	-23	11	-18
TTK	4/7(5)	27	12	61	73	18	13	31	28	43	71	21	23	16	6	16	5	5	6	-49	16	-18
NUF2	2/3(3)	26	6	39	45	9	16	24	20	33	53	43	41	20	24	31	9	3	3	-23	14	-6
**Sister Chromatid Cohesion Maintenance**																						
CDCA5	5/7(5)	42	15	47	62	21	9	31	40	28	68	10	12	12	4	8	4	6	7	-38	25	-19
PPP2R1A	2/7(5)	33	3	18	20	8	12	20	17	47	64	28	30	23	22	24	8	3	5	-5	14	30
SGOL1	3/7(5)	32	11	28	39	11	18	29	17	30	47	14	33	22	8	23	12	4	0	-10	6	2
**Chromosome Segregation**																						
ESPL1	7/7(5)	45	51	19	69	46	21	67	68	12	80	22	20	20	7	7	4	8	-5	2	17	-7
CDC16	2/3(3)	26	2	94	95	7	11	17	8	33	40	87	107	82	4	88	49	3	5	-83	6	-61
CDC26	2/3(3)	30	3	62	65	7	14	21	28	39	67	48	69	32	17	55	11	3	4	-48	25	-23
CDC27	2/3(3)	30	5	59	64	9	11	20	5	62	67	73	80	40	26	64	13	3	4	-49	0	3
**Cytokinesis**																						
ANLN	2/7(5)	38	3	23	26	3	3	6	13	9	22	57	55	53	42	52	41	3	1	-21	10	-14
PRC1	3/7(5)	34	9	24	33	13	6	19	41	19	60	38	34	16	25	27	6	4	4	-18	32	-5
RACGAP1	2/7(5)	29	12	9	21	12	13	25	46	32	77	15	19	15	12	14	3	3	0	4	34	23
ECT2	4/7(5)	28	27	9	36	30	6	36	42	12	54	15	16	18	10	10	8	5	2	-3	15	3
CHMP4B	2/3(3)	27	2	57	59	8	12	20	16	36	53	54	45	20	22	36	10	3	6	-45	14	-21
KIF23	2/7(5)	26	8	11	19	10	5	15	11	14	25	17	12	18	14	10	14	3	2	-6	3	3

Supplementary Figure S3: Genes essential to prevent EDR in HCT116 cells. HTS siRNAs are the number of positive siRNAs/ number of siRNAs tested in HTS (≥MAD value) from 'statistical analysis'. HTS ≥5N DNA is the fraction (%) of cells with ≥5N nuclear DNA content based on the median of the three siRNAs from DNA content analysis. Total cells are the number of cells counted relative to the number of cells in control (%) at end of experiment (3 days). 'Δ EDR' and 'Δ Apoptosis' are the differences ±ZVAD. They were calculated by subtracting the corresponding percentage for the TP53+ assay from the ZVAD assay. 'Δ EDR' and 'Δ Apoptosis' for the TP53 effect were calculated by subtracting the corresponding percentage for the TP53+ assay from the TP53- assay. Red indicates a reduction in EDR or Apoptosis, whereas blue indicates an increase. Gene names are from the Human Gene Nomenclature Committee (HUGO).
